# Psychoeducational group interventions for adults diagnosed with attention-deficit/ hyperactivity disorder: a scoping review of feasibility, acceptability, and outcome measures

**DOI:** 10.1186/s12888-024-05908-8

**Published:** 2024-06-20

**Authors:** Tatiana Skliarova, Henrik Pedersen, Åshild Holsbrekken, Sindre Andre Pedersen, Arthur Mandal, Carlos De Las Cuevas, Audun Havnen, Rolf Gråwe, Mariela Loreto Lara-Cabrera

**Affiliations:** 1 Department of Mental Health, Faculty of Medicine and Health Sciences, Norwegian University of Science and Technology (NTNU), Trondheim, Norway; 2 Division of Psychiatry, Nidaros Community Mental Health Center, St. Olavs University Hospital, Trondheim, Norway; 3 Library Section for Research Support, Data and Analysis, NTNU University Library, Norwegian University of Science and Technology (NTNU), Trondheim, Norway; 4 Vårres Regional User-Led Center Mid-Norway, Trondheim, Norway; 5 Department of Internal Medicine, Dermatology and Psychiatry, School of Medicine, University of La Laguna, San Cristóbal de La Laguna, Canary Islands Spain; 6 Department of Psychology, Norwegian University of Science and Technology (NTNU), Trondheim, Norway; 7 Department of Mental Healthcare, St. Olavs Hospital, Trondheim University Hospital, Trondheim, Norway; 8 Department of Mental Healthcare, Nidelv Community Mental Health Center, St. Olavs University Hospital, Trondheim, Norway

**Keywords:** Attention-deficit/hyperactivity disorder (ADHD), Feasibility, Patient education, Patient satisfaction, Psychoeducational intervention, Patient-reported outcomes (PROM), Scoping review

## Abstract

**Introduction:**

Although psychoeducational group interventions are increasingly used for adults diagnosed with attention-deficit/hyperactivity disorder (ADHD), a comprehensive review focused on the feasibility and acceptability indicators of these interventions remains lacking. Furthermore, although previous research has explored various aspects of psychoeducation for ADHD, such as its definition and approaches, limited research has focused on the synthesis for outcome measures and patients’ experiences related to these interventions. Therefore, this scoping review aims to map the existing evidence reported on psychoeducational group interventions for adults diagnosed with ADHD. The objective is to provide a comprehensive overview of feasibility indicators, acceptability, and outcome measures used in psychoeducational group interventions.

**Method:**

A comprehensive structured literature search on the topic was performed in seven bibliographic databases, and the resulting records were independently screened, and their data extracted by two reviewers. We followed the Preferred Reporting Items for Systematic reviews and Meta-Analyses Extension for Scoping Reviews (PRISMA-S) to ensure the transparency and rigor of this scoping review.

**Results:**

The searches yielded 7510 records. Eight studies met the inclusion criteria. These included studies were conducted in European countries and the United States. Among these, six studies used a randomized control design, one an open feasibility trial, and one a pre-post intervention design. All the studies reported some feasibility and acceptability indicators. While all the studies reported on the severity of symptoms of ADHD as an outcome measure, some also reported on outcomes related to psychological or mental-health problems, quality of life, changes in knowledge regarding ADHD, or the level of self-esteem, functioning, and impairment.

**Conclusion:**

This scoping review revealed that psychoeducational group interventions are generally acceptable for patients in terms of patient satisfaction with the group intervention. All included studies reported some feasibility indicators, with some reporting good attendance and relatively low dropout rates. Most studies reported positive effects on ADHD and mental health symptoms, suggesting that these interventions are beneficial for adults with ADHD. However, several gaps exist regarding the reporting on the feasibility indicators, acceptability, and outcome measures employed across studies.

**Supplementary Information:**

The online version contains supplementary material available at 10.1186/s12888-024-05908-8.

## Background

Attention deficit/hyperactivity disorder (ADHD) is a neurodevelopmental condition primarily characterized by long-term difficulties with symptoms of inattention, impulsivity, and hyperactivity [1, 2]. While ADHD is often recognized and diagnosed during childhood, its effects frequently extend into adulthood [3]. Furthermore, ADHD remains a significant concern in the adult population, with approximately 2.5% of adults worldwide estimated to be affected by this disorder [4]. In addition to presenting persistent challenges related to core symptoms, including hyperactivity, inattentiveness, and impulsivity, ADHD frequently co-occurs with other mental disorders [5–9] and is linked to difficulties in occupational performance [10–13]. These challenges can significantly affect working ability, psychosocial function, and educational achievement [12, 13]. Furthermore, adults with ADHD also report a diminished quality of life [14], substantial stigmatization [15], and fewer psychological protective factors, including self-management skills [16, 17]. For adults with ADHD, recent research and the current international clinical guidelines [18, 19] recommend a range of treatments encompassing pharmacological and nonpharmacological approaches [20–22]. Nonpharmacological approaches include psychosocial and educational strategies [20, 22, 23].

Psychoeducational interventions aim to improve patient attitudes (reflecting their perception of responsibility for their disease) and how they cope with their illness [24]. In psychiatric research, psychoeducation has gained recognition as a valuable adjunctive treatment for schizophrenia [25], psychosis risk [26], bipolar disorder [27], depression [28], and anxiety [29], and as a beneficial aid for caregivers and teachers of children with ADHD [30]. Psychoeducational group interventions have become increasingly important in managing chronic disorders [31]. Such interventions aim to provide information about the condition to adults newly diagnosed with mental disorders and offer crucial support. The primary focus of these interventions is to help patients learn and develop the skills necessary to independently manage their condition, adapt to, and live with their mental-health problems [32].

Recent studies have found that individuals diagnosed with ADHD want to learn more about various aspects of their diagnoses [33–36]. Psychoeducational programs enable individuals to acquire knowledge and gain a better understanding of their disorder and its associated challenges [37]. By delivering relevant information, these educational programs may also facilitate patients’ to accept their condition and alleviate negative emotions [37]. While individual psychoeducation is well implemented in clinical settings, in group-based interventions, the potential exists for further improvement in the delivery of information for adults newly diagnosed with ADHD. From user and mental healthcare perspectives, group-based intervention represents one method to provide information through shared learning [38]. In contrast to giving participants information individually, in a group setting, patients can learn from each other’s questions and coping strategies related to their condition. Furthermore, group-based psychoeducation makes peer support possible, and allows delivering comprehensive information and supporting more patients. Additionally, group-based interventions can appear to be promising, efficient interventions if they are feasible for mental healthcare services to deliver. Moreover, given that most psychoeducational programs in the field of mental health are complex group-based approaches [38–41], special attention should be focused on the acceptability of such programs.

Research on psychoeducational group interventions for adults with ADHD is still in its early stages, with no reviews focusing specifically on group-based interventions. A review conducted in 2016 [42], however, included three studies, of these only two focused on group-based approaches [43, 44]. Subsequently, a more detailed scoping review [45] was undertaken with a broader scope of identifying the characteristics of psychoeducational interventions tailored for adults with ADHD. This scoping review explored the various definitions and conceptualizations of “psychoeducation” within the context of these interventions. This later scoping review included a total of 10 articles published in English that specifically addressed psychoeducation for adults with ADHD [45]. Although the authors highlighted psychoeducational group interventions as a new approach to informing and educating adults diagnosed with ADHD, the review did not examine any feasibility or acceptability indicators for the intervention. Furthermore, although prior research has addressed various aspects of group-based approaches, the synthesis of the parameters used for outcome measures and patients’ experiences related to psychoeducational group interventions tailored to adults is limited. Some authors have claimed that patient-reported outcome measures (PROMs) are critical for promoting patient-centered care and evaluating patients’ views on their mental health, well-being, and functional status [46]. In addition, PROMs are included in clinical trials as indicators of the effectiveness of interventions, and they inform clinical practice and stakeholders about opportunities to improve the quality of mental-health treatment.

Given the nature of group-based psychoeducation and the limited number of studies reported in recent research [45], we conducted a scoping review [47, 48]. This study aims to map the existing evidence reported on psychoeducational groups interventions for adults diagnosed with ADHD. The objective is to provide a comprehensive overview of feasibility indicators, acceptability, and outcome measures used in psychoeducational group interventions, providing insight to researchers, clinicians, user organizations, and policymakers involved in group treatment and the informational support of these adults.

## Methods

Our study was conducted according to the recommended methodology guidelines for scoping reviews [47] and conforms with the Preferred Reporting Items for Systematic Reviews and Meta-Analyses Extension for Scoping Reviews (PRISMA-ScR) [49] and the frameworks for reporting the feasibility [50] and acceptability of health interventions [51].

Our review process commenced with the definition of objectives and the formulation of three clear and focused research questions to guide the review, in collaboration with user representatives from ADHD organizations. These questions aimed to identify and describe the feasibility and acceptability, as well outcome measures used in psychoeducational group interventions for adults diagnosed with ADHD. Additionally, we consulted with one user representative (AM) working in the field of ADHD and one (HH) working in the field of psychoeducation. User representatives helped define the objectives, develop the inclusion criteria, and ensure the accuracy and comprehensiveness of our review findings.

### Searches, eligibility criteria, and study selection

A medical research librarian (SAP) developed a rigorous search strategy. The search consisted of the three concepts “psychoeducation”, “ADHD” and “adults”. Relevant thesaurus terms and free text terms were searched within each concept and combined using the operator “OR”. Finally, all three concepts were combined using the operator “AND”.

The structured search strategy was conducted by a medical research librarian (SAP) and adapted to and run across the bibliographic databases MEDLINE, Embase, Web of Science, Cochrane Central, CINAHL, AMED, PsycINFO, and the register ClinicalTrials.gov. The search was last updated in all the databases on January 5, 2024, apart from PsycINFO, which was unavailable on that date and therefore last updated on June 7, 2022. The search interface for MEDLINE and Embase also changed from Ovid to EBSCOhost and Embase.com, respectively, in their last updates. Records captured in the literature search were imported into Endnote 20 reference management software, and duplicates were subsequently removed. Additional File 1 provides the detailed search strategy adopted for the various databases.

**Fig. 1:**
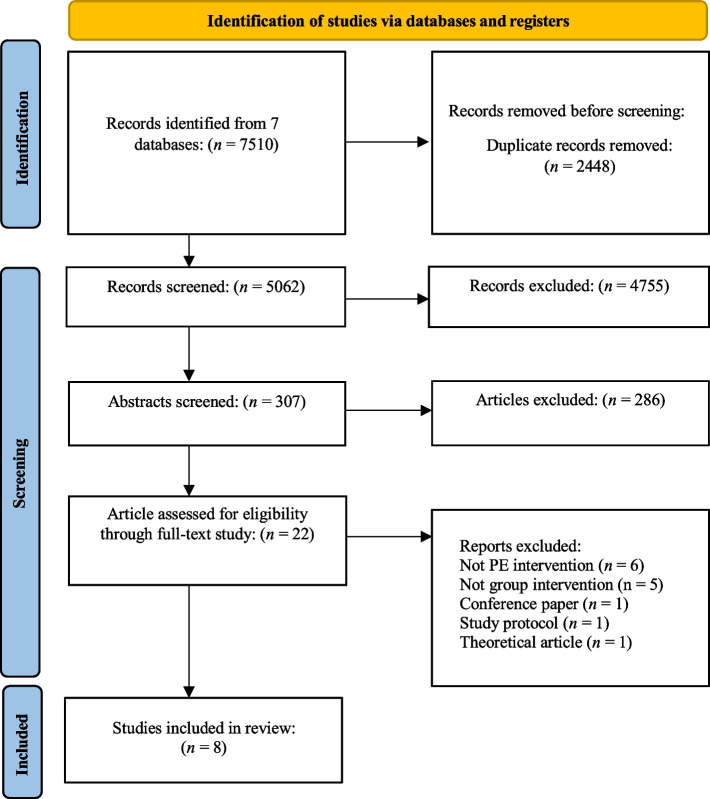
PRISMA flow diagram. Note: PE = Psychoeducation

The inclusion criteria encompassed peer-reviewed studies that reported findings from a psychoeducational group intervention program involving adults. Eligible studies included pilot studies, clinical studies, feasibility studies, and randomized controlled trials (RCTs). A study was considered for inclusion if it investigated the psychoeducational group intervention alone, in comparison to another treatment, or as a control group. The authors (TS, HP, AH, ÅH, and MLL-C) conducted a duplicate screening of titles and abstracts, including all articles that used the terms “ADHD” or “hyperkinetic disorder” and “psychoeducation” or “patient education”. Additionally, only studies with “adults” or “adulthood” mentioned in their title or abstract were included. The exclusion criteria encompassed studies that do not address the main research question or focus on non-psychoeducational interventions. In addition, grey literature, non-peer-reviewed articles, conference abstracts, protocols and theoretical articles were excluded. The study selection process is illustrated in the PRISMA flow diagram (Fig. 1).

### Data extraction and analysis

The data charting process used predefined tables, allowing us to extract, organize, and categorize the relevant information. Critical information related to the study characteristics, participant demographics, and intervention details was extracted independently by authors (TS, HP, RG, CdLC, and ÅH). Feasibility can be assessed using different feasibility indicators, such as the recruitment rate, retention rate, adherence, fidelity and engagement [50]. Acceptability reflects the extent to which providers or patients who participate in intervention consider it appropriate based on expected or experienced emotional and cognitive reactions to the intervention [51].

For data extraction regarding feasibility indicators, we included indicators as defined by previous studies [50, 52]. The extracted data included: the eligibility rate, recruitment rate, retention rate, drop-out rate, attendance of intervention’ sessions, fidelity, resources, statements regarding adaptation for the population and others. Concerning the acceptability indicators, we included data as defined by the authors if they explained how they measured acceptability (i.e., we employed patient’ satisfaction with the psychoeducational intervention as a measure, as assessed through qualitative or quantitative methods). The extracted acceptability indicators included: stakeholder acceptance, emotional and psychological effects of intervention, ethical considerations, patient satisfaction with the interventions, areas of concern, and contextual factors affecting acceptability. Last, we considered the outcome measurements reported by the authors. The extracted data included outcomes related to ADHD symptoms, skills, knowledge, quality of life, patient satisfaction, self-efficacy or self-esteem, functioning and impairment, and other reported outcomes.

Authors (TS, CdlC, and ÅH) independently extracted the feasibility criteria, acceptability, quality of reporting research (using the checklist items of Consolidated Standards of Reporting Trials, [CONSORT]) and outcomes used in psychoeducational group interventions. Disputes between the authors were resolved through discussion. Following the data charting, a narrative synthesis of the included studies was performed by summarizing the findings related to feasibility and acceptability indicators, as well as outcome measures used. This process aimed to identify patterns, gaps, and trends in the existing literature. The synthesis of the data was carried out based on predefined data-charting forms, and the final version of the extracted data was presented in the result tables and Additional File 2.

## Results

The searches yielded 7510 publications. The initial screening of titles and abstracts identified 22 potential studies for comprehensive full-text assessment. Of the 22 articles initially categorized as educational interventions for adults with ADHD, 14 were excluded. Due to the non-psychoeducational nature of the intervention after the full-text assessment, 10 studies were excluded. One of each of the following was excluded: a theoretical article, conference paper, study protocol, and psychoeducational chat-bot intervention. This exclusion left eight articles. Figure 1 illustrates the screening process and Additional File 4 provides information on the excluded publications.

### Characteristics of the studies

All included studies were conducted in one of five countries: two from Sweden [44, 53], three from Germany [54–56], one from Spain [43], one from Netherlands [57], and one from the USA [58]. The number of participants in these studies ranged from 27 to 179. Among the studies, seven included patients from outpatient settings [43, 44, 53–57], with two also incorporating significant others into the study [44, 53]. One study focused on college students diagnosed with ADHD [58].

Six studies were conducted in outpatient clinics [43, 44, 53–55, 57], while one took place in the student healthcare center [58], and one study was performed at a university clinic [56]. Six studies utilized a RCT design [43, 53–57], one employed a pre-post evaluation design [58], while the last was an open feasibility trial [44]. Of the six RCTs, two studies compared the effects of psychoeducational group interventions with Mindful Awareness Practices [54, 55], one study compared the effects of psychoeducation with the effect of cognitive-behavioral therapy [43], one study compared the effects of psychoeducation alone with those of a combination of psychoeducation and goal management training [57], one study compared psychoeducation with a waiting-list control group [53], and one study compared the same psychoeducational program in two groups delivered by two different methods, digital or pen-and-paper [56]. Only one RCT study [53] stated that the research followed the CONSORT reporting guidelines, and none reported using the Transparent Reporting of Interventions in Educational Research guidelines.

The duration of the group psychoeducational programs varied among the studies. Two studies reported a psychoeducational program consisting of 12 weekly sessions [43, 57], while five studies described eight weekly psychoeducational sessions [44, 53–56]. Additionally, one study reported six weekly group-based sessions [58]. It is noteworthy that two studies, both conducted by Hirvikoski et al., were based on the same psychoeducational program, PEGASUS [44, 53]. Table 1 presents more detailed information concerning the studies and interventions.

**Table 1:** Study characteristics

Study ID, author, year	Country, setting	Study design	Number of participants	Reporting guidelines	Sample size calculation	Intervention, description	Psychoeducation, number of sessions
Vidal, 2013 [43]	Spain, outpatient clinic	Randomized, prospective, controlled two-arm study	*n* = 32 participants: 15 in the psychoeducation group (PE) and 11 in the cognitive behavioral therapy group (CBT)	Not reported	Not reported	Group-based interventions. Both interventions (PE and CBT) were conducted by the same two clinical psychologists Psychoeducation focused on ADHD education and information, without practice skills or homework tasks. The CBT program focused on coping skills training and included limited psychoeducation	12 weekly sessions, over 3 months, 2 h each
Hirvikoski, 2015 [44]	Sweden, two outpatient tertiary psychiatric clinic	Open clinical feasibility trial	*n* = 108 participants: 51 with ADHD and 57 significant others (SO)	Not reported	Not reported	The PEGASUS program was described: a group psychoeducational program incorporating CBT, neuropsychology, and ADHD evidence Delivered by a group leader: psychiatrist, psychologist or occupational therapist, an individual with ADHD, social worker, an employee from a local employment service and from local municipality services The program focused on increasing knowledge about ADHD, treatment, support options in psychiatric care, and improving quality of life and relationship with significant others	eight weekly sessions 2.5 h each, 30 min break
Hirvikoski, 2017 [53]	Sweden, two outpatient tertiary psychiatric clinic	Pragmatic parallel group design, multicenter randomized controlled trial	*n* = 179 participants: 87 with ADHD, 92 SO Intervention group (IG): 48 ADHD, 49 SO, Control group (CG): 39 ADHD, 43 SO	CONSORT for reporting parallel groups randomized control trials	Was conducted: 0.80 power (at alpha level of .05) required approximately 50 participants with ADHD in each group	IG received manualized PEGASUS program in addition to treatment as usual CG received standard clinical services (not further specified) The PEGASUS groups included 10–15 adults with ADHD + at least as many SO Other information: the intervention is the same as in Hirvikoski et al.’s, 2015 study	8 weekly sessions 2.5 h each, 30 min break
In de Braek, 2017 [57]	Netherlands, outpatient setting	Randomized controlled trial	*n* = 27 IG: Goal Management Training (GMT) in combination with PE, *n* = 12 CG: PE, *n* = 15	Not reported	Not reported	IG received GMT in combination with PE. The intervention aimed to improve participants’ planning and strategy skills (GMT) and PE CG received the same psychoeducational sessions as IG, but did not receive GMT	12 weekly sessions 1.5 h each, 1 individual session and 11 group sessions
Hoxhaj, 2018 [54]	Germany, outpatient setting	Randomized controlled trial with a factorial design	*n* = 81 medication-free adult ADHD patients PE, *n* = 40 MAP, *n* = 41	Not reported	Was conducted: To detect effect size of *d* = 0.6, a sample of 72 participants were required. 82 randomized patients were planned	One group received a group-based PE program, the other group received MAP The groups were conducted by trained psychotherapists and experts in CBT and mindfulness-based cognitive therapy (MBCT) MAP group sessions and homework: daily meditation and mindfulness exercises PE: provided information about ADHD in adulthood and the activation of organizational skills, stress management techniques, and self-esteem	8 weekly sessions of 2.5 h
Bachmann, 2018 [55]	Germany, outpatient setting	Randomized control fMRI study	*n* = 74, 37 in each group, PE and MAP	Not reported	Not reported	One group received group-based PE program; the other group received MAP Other information: the intervention was the same as in Hoxhaj et al.’s, 2018 study	8 weekly sessions of 2.5 h each,
Hartung, 2022 [58]	USA, university campus, two psychology training clinics	Pre-post design	*n* = 30 students from two universities	Not reported	Was conducted: 27 participants were necessary to achieve adequate power (0.80) to detect a medium effect size (*d* = 0.50) using matched pairs *t*-tests	Group and individual sessions of PE. PE included information about ADHD, treatment options, and coping (comprising planning and academic skills) Delivered by advanced graduate student therapists and licensed clinical psychologists The intervention was designed specifically for college students with ADHD. The intervention was focused on organizational, time management, and planning skills and demands of college students	6 group sessions, 3 individual sessions All sessions were held across 6 weeks. Only 2 sessions were defined as psychoeducation
Selaskowski, 2022 [56]	Germany, university clinic	Randomized controlled trials	*n* = 60 participants, 30 in smartphone-assisted PE (SAP), 30 in brochure-assisted PE (BAP)	Not reported	Not reported	One group received SAP, the other group received BAP The groups were led by experienced therapists The content of the group intervention was identical in both groups, only the work materials and format were different: SAP in digital form, BAP in pen-and-paper form The program consisted of psychoeducation content, homework assignments, and a content quiz	8 weekly group-based psychoeducational sessions

*ADHD* Adult Attention Deficit/Hyperactivity Disorder, *BAP* Brochure-assisted psychoeducation, *CBT* Cognitive behavioral therapy, *CG* Control group, *CONSORT* Consolidated Standards of Reporting Trials, *GMT* Goal Management Training, *IG* Intervention group, *MAP* Mindful Awareness Practices, *MBCT* Mindfulness-Based Cognitive Therapy, *PE* Psychoeducation, *SAP* smartphone-assisted psychoeducation, *SO* Significant others, *N* Total number of participants

Regarding demographic data, most of the studies [43, 44, 53, 54, 56, 57] reported information on patients’ age, gender, level of education, some studies in addition reported ADHD subtype and employment status [43, 44, 53, 54, 56]. Some studies provided details on the number of years since ADHD diagnosis [44, 53] and the presence of co-morbid psychiatric disorders [53–56] (Table 4).

### Feasibility indicators

Regarding the PEGASUS intervention, the authors pre-defined acceptable levels of feasibility indicators, such as a dropout rate of no more than 25% and treatment completion of at least 50% of attended sessions [44, 53]. Only two studies reported the eligibility rate [54, 56], which was 52% and 28%, respectively. More information is presented in Table 2.

**Table 2:** Feasibility indicators

**Characteristics**	Vidal, 2013 [43]	Hirvikoski, 2015 [44]	Hirvikoski, 2017 [53]	In de Braek, 2017 [57]	Hoxhaj, 2018 [54]	Bachmann, 2018 [55]	Hartung,2022 [58]	Selaskowski, 2022 [56]
Dates of recruitment (YYYY.MM)	-	-	2012.03 to 2013.12	-	2012 to 2013	-	-	2019.03 to 2020.11
Eligibility rate^1^	-	-	-	-	52% (103 out of 200 were eligible)	-	-	28% (67 out of 236 were eligible)
Recruitment rate^2^	80% (32 out of 40)	Patients: 96.2% (51 out of 53); SO: 89% (57 out of 64)	IG, patients: 95.8% (46 out of 48); SO: 93.9% (46 out of 49) CG, patients: 94.9% (37 out of 39); SO: 95,3% (41 out of 43)	-	78.6% (81 out of 103 invited participants were randomized)	-	-	94% (63 out of 67)
Session attendance	-	-	Patients attended 86% of the sessions; SO attended 79% of the sessions	-	-	-	Attendance at mandatory group sessions was 87%	SAP: mean = 6.7 sessions BAP: mean = 6.9 sessions
Retention rate at post intervention	93.75% (30 out of 32)	Patients: 84.3% (43 out of 51) SO: 73.7% (42 out of 57)	IG, patients: 93.8% (45 out of 48); SO: 89.8% (44 out of 49) CG, patients: 87.2% (34 out of 39); SO, 83.7% (36 out of 43)	IG: 83% (10 out of 12) CG: -	PE: 90% (36 out of 40); MAP: 95% (39 out of 41)	PE: 73% (27 out of 37); MAP: 86.5% (32 out of 37)	100%	SAP: 86.7% (26 out of 30) BAP: 66.6% (20 out of 30)
Retention rate at follow-up	81.25% (26 out of 32)	At 6-months follow-up Patients: 80% (41 out of 51), SO: 70% (40 out of 57)	At 3-months follow-up IG, patients: 87.5% (42 out of 48); SO: 73.5% (36 out of 49) CG, patients: 79.5% (31 out of 39); SO: 72.1% (31 out of 43)	At 12 weeks follow-up IG: 66,7% (8 out of 12) CG: - At 24 weeks follow-up: IG: 50% (6 out of 12) CG: -	At 6-months follow up PE: 80% (32 out of 40); MAP: 78% (32 out of 41)	-	-	-
Drop-out rate	6% (2 out of 30)	Patients: 15.6% (8 out of 51) SO: 29.3% (15 out of 57)	IG, patients: 4.2% (2 out of 48), SO: 8.3% (4 out of 48) CG: patients: 5.1% (2 out of 39), SO: 4.7% (2 out of 43)	IG: 17% (2 out of 12) CG: -	PE: 10% (4 out of 40); MAP: 4.9% (2 out of 41)	PE: 27%, 10 out of 37 MAP: 13.5%, 5 out of 37	0	Total: 27% (17 out of 63 randomized) SAP: 13.3% (4 out of 30) BAP: 33.3% (10 out of 30)
Evidence of reliability and validity of PROM and PREM in the target population^3^	Partially reported	Only one scale	Partially reported	Partially reported	Partially reported	Partially reported	Partially reported	Partially reported
Resources used	Two clinical psychologists were trained to adhere to the content of each treatment	Two group leaders were provided with workbooks, lecture materials, and other informational materials	Workbooks for course leaders and handbooks for participants. Lecture material such as PowerPoint presentations were provided for group leaders	Two clinical neuropsychologists conducted the intervention, one blinded neuropsychologist conducted the clinical assessment	Two therapists conducted the intervention	Intervention was given by health professionals	Graduate students under the supervision of clinical psychologists	Intervention was given by two therapists; paper materials were used in the BAP group
Risk assessment	-	-	-	-	-	-	-	-
Key stakeholders included	-	-	-	-	-	-	-	-
Statements regarding adaptation for the population	-	The intervention was developed especially for adults with ADHD	The intervention was developed especially for adults with ADHD	-	-	-	The intervention was developed especially for college students with ADHD	-
Implementation	-	-	-	-	-	-	-	-
Potential barriers	-	-	-	-	-	-	-	-
Fidelity	Partially reported	Partially reported	No significant deviations from the intended PE program were noted	Partially reported	To ensure fidelity to the manual and quality assurance of both group programs, all therapy sessions were video recorded	Partially reported	Partially reported	Partially reported
Other issues reported	-	-	In an outpatient psychiatric context, scarce resources (such as sufficient time) may hamper the implementation of new methods	-	Authors reported the PE was easier to implement, while the MAP groups necessitated specially trained therapists	Insufficient behavioral responses during conducting fMRI or	-	Participant needed access to a smartphone with Android OS

Not reported, *ADHD* attention deficit/hyperactivity disorder, *CG* control group, *fMRI* functional magnetic resonance imaging, *IG* Intervention group, *MAP* Mindfulness training, *PE *Psychoeducation, *PREM* Patient reported experiences, *SO* significant other

^a^Eligibility rate defined as the number of participants eligible divided by n of participants screened (n or %)

^b^Recruitment rate defined as n of participants accepting the invitation, divided by n of participants invited

^c^Authors reported evidence of reliability and validity of measures in the target population; or there is evidence of psychiatric properties in the target population

The recruitment rate was reported in five studies, ranging from 78.6% [54] to 96.2% [44]. All studies reported a retention rate, with the lowest rate being 66.6% [56], and the highest being 100% [58]. Session attendance was reported in three studies [53, 56, 58], with two at 86% and 87% [53, 56], and the last study reported a mean attendance of 6.7 to 6.9 out of eight group sessions [58].

All studies reported dropout rates from the interventions, with the highest dropout rate 27% [55, 56] and the lowest 0% [58]. Only one study reported an assessment of the feasibility of the interventions regarding the implementation cost [58]. Additional information is presented in Table 2.

### Acceptability indicators and patient satisfaction

Acceptability was operationalized in different ways across the studies (qualitative and quantitative). The Evaluation Questionnaire was used to measure satisfaction with the intervention in two studies [44, 53] and reached at least mean score equal 2.5 (the scale range is from 0 to 4) in one study [44], and mean score equal 3 (in 7 out of 8 occasions) in the other study [53]. In one of these studies [44], patient satisfaction was reported using patients’ rates on the course and session evaluation form and reached mean value equal 2.21 (*SD* = 0.72, the range of the score is from 0 to 3). Table 3 presents more detailed information about the acceptability characteristics of the studies.

**Table 3:** Acceptability indicators

Study ID, author, year	Tools and methods for measuring acceptability	Stakeholder acceptance	Emotional and psychological effect of the intervention	Ethical considerations	Acceptability and/or patient satisfaction with the group intervention	Areas of concern	Contextual factors affecting acceptability
Vidal, 2013 [43]	Not reported	Not reported	Not reported	Yes, this study was approved by the ethics committee of the hospital, and all the participants signed an informed consent document	Not reported	Not reported	Not reported
Hirvikoski, 2015 [44]	Modified version of the evaluation form, rating the course, session evaluation form (SEF) to get feedback	Not reported	Not reported	Yes, the study was approved by the Regional Ethics Committee of Stockholm (2009/824–31/3)	Treatment satisfaction was good, patients with ADHD showed more willingness to participate in the future than SO	Not reported	Not reported
Hirvikoski, 2017 [53]	Modified version of the evaluation questionnaire	Not reported	No adverse events (any inconvenience that a participant reported) or serious adverse events (anything that required inpatient hospitalization) were judged to be related to the program per se	Yes, the study was approved by the Regional Ethics Committee of Stockholm in 2012 (2012/422–31/3), and all participants gave their informed consent	The participants reported generally good treatment satisfaction	Not reported	For resource saving reasons, psychoeducational groups were relatively large: 10–15 adults with ADHD and at least as many SO
In de Braek, 2017 [57]	Not reported	Not reported	Not reported	Not reported	Not reported	Not reported	6–8 participants in each group, to ensure that the training functioned optimally
Hoxhaj, 2018 [54]	Not reported	Not reported	Not reported	Yes, the study was approved by the local ethics committee of the University of Freiburg	Not reported	Not reported	Not reported
Bachmann, 2018 [55]	Not reported	Not reported	Not reported	Yes, the study was approved by the local ethics committee and registered in the Current Controlled Trials database (ISRCTN12722296)	Not reported	Not reported	Not reported
Hartung, 2022 [58]	Qualitative feedback from the participants	Not reported	Not reported	Not reported	Participants reported satisfaction with the intervention, both during and after completion, suggesting that it is easily tolerated	30% of students prescribed ADHD medication misuse it at some point in their college careers, stigmatization is a concern	Not reported
Selaskowski, 2022 [56]	Not reported	Not reported	No adverse events or unintended consequences were reported	The study was approved by the ethics committee of the University of Bonn (232/18), and written consent was obtained	Not reported	Not reported	The intervention was addressed only to the participants familiar with smartphones

*ADHD* attention deficit/hyperactivity disorder, *SO* significant other

### Outcomes measures used to evaluate the interventions

Regarding ADHD symptoms, four studies [43, 54, 55, 58] used Conners’ Adult ADHD Rating Scale (CAARS [59]), and two studies [44, 53] used Adult ADHD Self-Report Scale (ASRS [60]), while four studies [44, 53, 54, 56] used the Wender Utah Rating Scale (WURS [61]) to assess the diagnosis of ADHD at baseline. One study [56] used the Integrated Diagnosis of ADHD in Adulthood (IDA-R [62]) and one study [57] used the Clinician’s Interview-Based Impression of Severity (CIBIS [63]) and Symptom Check List–90 (SCL-90 [64]). Five studies reported depression and anxiety symptoms [43, 44, 53, 54, 56] using different scales. Additionally, the Perceived Stress Scale (PSS [65]) was used to measure stress Hirvikoski et al.’s, (2015) study [44]. Quality of life or global life satisfaction was reported, using different measurement scales. For detailed information, see Table 4.

**Table 4:** Outcomes measures used to evaluate the interventions

**Study ID, author, year**	**Variables reported at baseline**	**Outcomes reported at baseline**	**Reported at post-intervention or follow-up**	**ADHD symptoms**	**Skills**	**Measures of knowledge**	**Self-rated scales not validated in ADHD**	**Quality of life or well-being**	**Patient satisfaction**	**Self-efficacy or self-esteem**	**Functioning and impairment**	**Other outcomes reported**
Vidal, 2013 [43]	Age, gender, marital status, level of education, work status, ADHD subtype, medication	Primary: ADHD-RS^a^; CAARS-S^a^; CGI-S Secondary: BDI; STAI; QLESQ^a^	Same as baseline at post intervention	ADHD-RS^a^; CAARS-S^a^; CGI-S	No	No	BDI; STAI; CGI-S	QLESQ^a^	No	No	No	No
Hirvikoski, 2015 [44]	For patients: age, gender, ADHD subtype, years diagnosed with ADHD, treatment, work status, education, Full-scale IQ, WURS-25*, ASRS*	ADHD 20 Questions (knowledge quiz, 20 true/false items) QAFM; BDI; BAI; PSS; RSE; AAQoL^a^	Same as baseline at post intervention and 6 months follow-up	WURS^a^; ASRS^a^	No	ADHD 20 Questions (knowledge quiz, 20 true/false items)	QAFM BDI; BAI; PSS; RSE	AAQoL^a^	Treatment satisfaction; Rating course from 0 to 4; Session evaluation form at the end of each course session (completed anonymously) RSE	RSE	No	QAFM
Hirvikoski, 2017 [53]	Patients: age, gender, ADHD subtype, years since diagnosed, early psychiatric care, current treatment, work status, education SO: age, gender, work status, education, relation to the patients	Primary: ADHD 20 Questions, (knowledge quiz, 20 true/false items) Secondary: SWLS; HADS; QAFM; RSE; SO: BAS	Same as baseline at post-intervention and follow-up	WURS^a^; ASRS^a^	No	ADHD 20 Questions, (knowledge quiz, 20 true/false items)	HADS; SWLS; RSE QAFM	SWLS	Session evaluation form at the end of each course session (completed anonymously); Modified evaluation scale focusing on gained knowledge	RSE	No	QAFM; BAS; Adverse effects
In de Braek, 2017 [57]	Age, gender, education (years), use of the medication	CIBIS/C; SCL-90; CFQ; Zoo Map (BADS)	Same as baseline	CIBIS CIBIC SCL-90	Zoo Map (BADS)	No	CIBIS/C; SCL-90; CFQ	No	No	No	CFQ	No
Hoxhaj, 2018 [54]	Age, gender, ADHD subtype, marital status, education, IQ, employment status, co-morbidities WURS-k*	Primary outcomes: CAARS^a^ inattention/memory subscale Secondary outcomes: CAARS^a^ subscales (observer-rated and self-rated); BDI; BSI; SF-36; FFMQ	Same as baseline	CAARS^a^	FFMQ	No	BSI; BDI; BSI; SF-36; FFMQ	SF-36	No	No	No	FFMQ
Bachmann, 2018 [55]	Age, education, gender, ADHD subtype, medication, comorbid psychiatric disorder, current and lifetime	CAARS^a^ self- and blind observer measurement of brain activation in the frontoparietal regions and basal ganglia; working memory task during fMRI;	Same as baseline	CAARS^a^	Working memory task during fMRI	No	No	No	No	No	No	fMRI
Hartung, 2022 [58]	Age, gender, race, level of education, previous diagnosis of ADHD	DSM-5; WFIRS; CAARS^a^; OTMP	Same as baseline	DSM-5; CAARS^a^	OTMP	No	OTMP; WFIRS	No	Qualitative	No	WFIRS; OTMP	No
Selaskowski., 2022 [56]	Age, gender, education level, employment status, ADHD subtype, pharmacological treatment, comorbid disorders	IDA-R^a^; BDI-II; WFIRS; MWT-B; WURS-k^a^; Content quiz	IDA-R^a^; BDI-II; WFIRS; Content quiz	DSM-5; IDA-R^a^	No	Content quiz at the end of each module	MWT-B; BDI-II; WFIRS	No	No	No	WFIRS	MWT-B

^a^Authors reported the scale was validated among ADHD

*AAQoL* Adult Attention Deficit/Hyperactivity Disorder Quality-of-Life, *ADHD-RS* *ADHD*–Rating Scale, *ASRS* Adult ADHD Self-Report Scale, *BAI* Beck Anxiety Inventory, *BAS* Burden Assessment Scale, *BDI* Beck Depression Inventory, *BSI* Brief Symptom Inventory, *CAARS* Conners Adult ADHD Rating Scale, Long Version, *CAARS-S* Conners Adult ADHD Rating Scale, Shor Version, *CFQ* Cognitive Failures Questionnaire, *CGI-S* Clinical Global Impression–Severity Scale, *CIBIS/C* Clinician’s Interview-Based Impression of Severity/Change, *FFMQ* Five-Facet Mindfulness Questionnaire, *HADS *Hospital Anxiety and Depression Scale, *IDA-R* Integrated Diagnosis of ADHD in Adulthood, *MWT-B* Mehrfachwahi-Wortschat Test, *OTMP* Organizational Time Management and Planning, self-reported, *PSS* Perceived Stress Scale, *QAFM* Questions about family members, *QLESQ* Quality of Life Enjoyment and Satisfaction Questionnaire, *RSE* Rosenberg’s Self-Esteem, *SF-36* 36-Item Short Form Health Survey, *SCL-90* Symptom Check List-90, *STAI *State-Trait Anxiety Inventory, *SWLS* Satisfaction With Life Scale, *WFIRS* Weiss Functional Impairment Rating Scale, *WURS-25* Wender Utah Rating Scale, *Zoo Map (BADS)* Zoo Map for the Behavioral Assessment of the Dysexecutive Syndrome

### Reported main findings

All studies exhibit significant improvements in one or more outcome measures. Six of eight studies reported improvement in ADHD symptoms over time [43, 54–58], but only two RCTs displayed improvement in symptom domains between groups [56, 57]. Two studies reported improvement in anxiety and depression symptoms [43, 44], one reported improvement in the subjective stress level over time [44], and one reported improvement in ADHD-related impairment [53, 58]. Two studies indicated improvement in the level of knowledge regarding ADHD [44, 53], and one indicated improvement in self-esteem after intervention [43]. Three studies reported improvement in other outcomes. Bachmann et al. (2018) [55] found improvement in task performance; Hartung et al. (2022) [58] reported improvement in organization, time management and planning skills; finally, Selaskowski et al. (2022) [56] reported improvement homework compliance after the intervention. Only one study reported significant improvement in the quality of life over time [43].

## Discussion

The primary goal of this scoping review was to map the existing evidence reported on psychoeducational group interventions for adults diagnosed with ADHD. The objective was to provide a comprehensive overview of feasibility indicators, acceptability, and outcome measures used in these interventions. We identified eight studies that satisfied the inclusion criteria.

Although all included studies reported some feasibility indicators, and only one study was characterized as a feasibility study [44], the results were heterogeneous and problematic to compare. For instance, recruitment was primarily presented through a flow chart, making it challenging to assess the various feasibility criteria. Few studies made any predefined assumptions about feasibility [44, 53].

Hirvikoski et al. [44] applied their inclusion criteria to sample selection from the general ADHD clinical population. This type of approach is useful because the high frequency of psychiatric and somatic comorbidities in adult ADHD patients [66] could result in strict inclusion criteria that exclude a large portion of this population and thereby limit the potential for large-scale implementation of the intervention. Although the reported interventions had broad inclusion criteria, some studies used concurrent psychopathology and medication use as exclusion criteria [43, 54, 57]. Data on the recruitment period and eligibility rate were mostly lacking, making it challenging to identify crucial feasibility characteristics, such as the duration of the study or the degree of suitability of the intervention for ADHD patients.

Three studies reported high attendance rates [53, 56, 58]. Factors like scheduling conflicts could be potential barriers to programming the implementation [67]. Only the study by Hartung et al. [58] reported this barrier and tailored its intervention to the participants’ needs. Moreover, their study was the only one to consider the cost of the study for the participants. Their study reported the lowest drop-out rate and highest attendance rate, even though indirect and direct costs can represent barriers to enrollment and participation in psychoeducational programs [67]. Attendance at the sessions may reflect patients’ adherence to the treatment and thus provide information about how critical the intervention is for the participants [51]. The reporting on this outcome, however, was generally sparse and variable. Only two studies [44, 53] reported predefined criteria for attendance rates. Considering that these interventions are in the initial stages of development, future studies on the development and evaluation of psychoeducational group programs should prioritize reporting attendance measures. The scarcity of data on the recruitment period and eligibility rate, coupled with the lack of standardization of feasibility indicators, necessitates further research.

Only the study by Hirvokiski et al. [53] stated that the research followed the CONSORT reporting guidelines for an RCT study (Additional File 2). The other studies did not indicate the use of guidelines or recommendations for RCT or feasibility research in their studies. Compliance with guidelines increases the transparency of research and promoting deeper and more critical analysis by other researchers [68]. Additionally, only three studies reported sample size calculations [53, 54, 58], although this is a vital element when conducting clinical trials to demonstrate significant differences [69]. Thus, future studies should follow and indicate compliance with reporting guidelines to provide a template for the intervention description and replication framework [70]. In addition, future studies should prioritize conducting more rigorous research regarding determining appropriate sample sizes and reporting recruitment feasibility challenges to facilitate a better understanding of the feasibility and effects of such interventions.

Assessing acceptability and patient satisfaction is vital in developing new interventions in clinical settings [51]. This review reveals that acceptability, defined as patient satisfaction with the group intervention or the emotional and psychological effect of the intervention, went unreported in most interventions. Only three studies used evaluation questionnaires or other measures to assess the acceptability of an intervention [44, 53, 58]. Two studies reported patient satisfaction as ad hoc measures [44, 53], and one employed a qualitative approach to assess satisfaction [58].

Another identified challenge is the use of modified and non-validated scales to report patient satisfaction. Standardization in reporting acceptability and patient satisfaction is crucial for developing and improving the content of interventions [51]. This aligns with the findings of previous studies that little attention has been paid to performance measures and the assessment of patient’ viewpoints [71]. Furthermore, using reliable outcome measures to report patient experiences can enhance practice, making the results meaningful in and of themselves and facilitating inter-study comparisons [72]. In addition, the use of validated scales and tools to measure participants’ experiences provides mental-healthcare decision-makers with the necessary information regarding meaningful outcomes for patients [73]. Therefore, the results of the current study strongly recommend using standardized and validated tools to measure the acceptability of and patient satisfaction with these psychoeducational group programs. Nevertheless, the findings should be interpreted with caution, given the methodological limitations and limited number of studies reporting on satisfaction.

The acceptability level of the interventions among mental-healthcare providers, stakeholders, and patient representatives involved in the interventions was not reported in the included studies. In addition, only the study by Hartung et al. reported the adaptivity criteria for participants’ needs [58]. This pattern may reflect little collaboration or a lack of involvement or support from users, mental-healthcare professionals, and clinicians, which requires further exploration. This finding also highlights that mental-healthcare services and funding should be involved in developing and evaluating such interventions [31]. Adapting psychoeducational group interventions can be essential for global mental health equity. Research in this area could lead to innovative, cost-effective solutions, ensuring that effective patient education for ADHD care is accessible to patients with economic constraints. Future studies should focus on developing sensitive and culturally acceptable interventions to meet patient needs (sociocultural or demographic).

According to guidelines for reporting the acceptability of healthcare interventions [51], studies should measure the emotional and psychological effect of the intervention. As only one study measured the potential emotional stress or adverse effects of the interventions [53], future studies should focus on assessing these adverse effects, as well as the emotional and psychological effects that the intervention might cause.

The findings of this scoping review underscore the positive influence of psychoeducational group interventions on adults with ADHD, particularly in improving ADHD symptoms. These results align with those of Nimmo-Smith et al. (2020) [20], Oliveira et al. (2018) [74], and Montoya et al. (2011) [30], who found that non-pharmacological interventions improve ADHD symptoms among children [30] and adults with ADHD [20, 74]. Moreover, the positive outcomes reported in this scoping review align with previous research highlighting the effectiveness of psychoeducational interventions in other psychiatric conditions. Studies on various conditions, such as schizophrenia [25], psychosis risk [26], bipolar disorder [27], depression [28], and anxiety [29], have similarly demonstrated improvement in patient knowledge, and symptom management following psychoeducational intervention. However, while all studies assessing patient-reported outcomes reported improvement in one or more outcomes, the findings also indicate the variability in outcome measures used.

All the included studies reported a variety of outcome measures, such as knowledge about ADHD, relationships with family members, psychological well-being, cognitive failures, ADHD core symptoms, and symptoms of anxiety and depression. Overall, four studies directly addressed quality of life [43, 44, 53, 54]. Only one study, by Hirvikoski et al. [44], used a specialized scale to measure the quality of life of adults with ADHD, while other studies used more general questionnaires for measuring quality of life, thus limiting the data-pooling potential for meta-analyses in future studies. Furthermore, as the studies used diverse outcome measures and operationalized quality of life and well-being differently, further understanding the effects of these psychoeducational group interventions on the quality of life is challenging. Consistently measuring and operationalizing this outcome in future research is necessary.

According to previous studies, knowledge [35], and self-efficacy [31] are important factors related to educational interventions. We did also find, however, that measuring these outcomes was rarely reported. None of the studies reported any effects on self-efficacy. Only three studies in this review measured knowledge gain, using a nonvalidated scale [44, 53, 56]. Although measuring knowledge gain is a critical outcome for improving treatment enrolment and adherence [75], barriers exist to measuring and interpretating knowledge gain [76]. To overcome these obstacles, we recommend using standardized validated PROMs to measure outcomes.

Previous studies have highlighted the value of group interventions by providing a forum through which participants can share their experiences with others, thereby increasing social support [31]. This aspect is particularly vital in addressing the social isolation and misunderstanding that many adults with ADHD face. The group setting provides a safe space for patients to connect with others who understand their struggles, fostering a supportive network that extends beyond the clinical environment. Still, studies measuring the social isolation and the stress levels in everyday life are lacking, highlighting the need for more research in this field.

The wide range of identified outcome measures investigating the effects of mental health outcomes found in this scoping review highlights the need to include similar scales in future studies. This approach would allow for the comparison and replication of findings and facilitate future systematic reviews and meta-analyses. When standard information on mental health outcomes becomes available, it will be possible to recognize and pinpoint the effects of the interventions, understand the mechanisms of improvement, and identify specific approaches that should be avoided, improved, or preferred in the context of ADHD.

Opportunities for improvement based on the available evidence on outcome measures used in the included studies were identified. Some studies used self-developed measures [44, 53, 56] or patient-reported outcomes without referring to their psychometric properties [44, 58]. Some studies also used patient-reported scales to measure anxiety and depression, but these scales have not been validated for the adult ADHD population [43, 44, 53, 54, 56]. The evaluation of psychoeducational interventions requires adaptation and validation of PROMs and self-rated scales, considering these methodological limitations. Hence, these findings highlight the need for more studies using psychometric methods that employ validated self-reported scales specifically targeting ADHD. These adaptations and validations can add further research value, yielding meaningful pooled results in future systematic reviews and paving the way to compare and determine the effectiveness of psychoeducational interventions in the emerging mental-health field regarding the treatment of adults with ADHD.

## Strengths and limitations

This scoping review was based on seven databases. It provides, for the first time, an overview of the feasibility and acceptability of psychoeducational group interventions for adults diagnosed with ADHD while offering an overview of the applied outcome measures. This review can assist researchers and guide future work on intervention development and research in this field. Although the findings provide knowledge to support health policymakers and clinicians with a broad understanding of psychoeducational group interventions for adults diagnosed with ADHD, the findings are limited to a few studies, indicating that this research is still in its early developmental stages. Including special subject databases such as PsycINFO is generally recommended when the review topic directly touches the primary focus of a database. The exclusion of PsycINFO from the most recent update of the literature search for this study means that some recent studies, unique to this database, could have been overlooked. This therefore represents another potential limitation in this study.

The inclusion criteria restricted the selection of studies to only those in English, limiting the findings and potentially introducing selection bias. Another limitation arises from the exclusive focus on group-based psychoeducational programs. Furthermore, most studies were conducted in outpatient settings with adult patients with ADHD, limiting the generalizability of their results to other populations. Additionally, while this study primarily focused on the acceptability of patients who received group psychoeducational programs, it does not assess the acceptability from the perspective of providers and stakeholders, which represents another limitation.

In addition, most of the included studies were from Europe, limiting the representativeness of the findings. Thus, future research addressing a more diverse population is necessary. Adapting these psychoeducational group interventions to lower-resource settings can be essential for global mental health equity. Research in this area could lead to innovative, cost-effective solutions, ensuring that effective patient education and ADHD care are accessible to patients with economic constraints.

Finally, we identified several outcome measures, but the findings are limited by the lack of standardize measurement of outcomes for feasibility, acceptability, and patient-reported outcomes and experiences. Educational group interventions can significantly affect the improvement of patient knowledge regarding the disorder [35], adherence, social and occupational functioning, and clinical outcomes [75]. Future studies could benefit from measuring such outcomes. Moreover, there are other outcomes on which educational group interventions can have an impact on, promoting peer support, coping strategies, and self-management outcomes (e.g. level of patient activation and self-efficacy [31]), that should be evaluated in future studies.

## Conclusion

This scoping review revealed that psychoeducational group interventions are generally acceptable for patients in terms of patient satisfaction with the group intervention. All included studies reported some feasibility indicators, with some reporting good attendance and relatively low dropout rates. Most studies reported positive effects on ADHD and mental health symptoms, suggesting that these interventions are beneficial for adults with ADHD. Several gaps exist, however, regarding the reporting on feasibility indicators, acceptability, and outcome measures used across studies. Some studies have only partially followed standard reporting guidelines. Patient-reported outcomes were consistently incorporated into the existing studies. Patient-reported experiences regarding stress or the level of self-efficacy were lacking. Some critical aspects of acceptability, such as acceptability of providers and stakeholders, were missing. In addition, some studies used patient-reported outcomes that were not validated in adults diagnosed with ADHD. Future research should aim to fill these gaps regarding the lack of standardized feasibility criteria and limited reporting on acceptability and patient satisfaction.

### Supplementary Information

Additional file 1.

Additional file 2.

Additional file 3.

Additional file 4.

Additional file 5.

## Data Availability

All data generated or analysed during this review are included in this published article and its supplementary information files.

## Abbreviations

AAQoL: Adult attention deficit/hyperactivity disorder quality-of-life scale
ADHD: Attention deficit hyperactivity disorder
ASRS: Adult ADHD Self-report scale
BAI: Beck anxiety inventory
BDI: Beck depression inventory
BSI: Brief symptom inventory
CAARS: Conners’ adult ADHD rating scale
CFQ: Cognitive failures questionnaire
CGI-S: Clinical global impressions scale -severity
CIBIS/C: Clinician’s interview-based impression of severity/ change
CONSORT: Consolidated standards of reporting trials
FFMQ: Five facet mindfulness questionnaire
fMRI: Functional magnetic resonance imaging
GMT: Goal management training
HADS: Hospital anxiety and depression scale
IDA-R: Integrated diagnosis of adhd in adulthood
MWT-B: Mehrfachwahi-wortschat-test
PE: Psychoeducation
PREM: Patient reported experiences
PRISMA-ScR: The preferred reporting items for systematic reviews and meta-analyses extension for scoping reviews
PROM: Patient-reported outcome measure
PSS: Perceived stress scale
QAFM: Questions about family members
QLESQ: Quality of life enjoyment and satisfaction
RCT: Randomized controlled trial
RSE: Rosenberg’s self-esteem scale
SCL-90: Symptom check list-90
SF-36: 36-Item short form health survey
STAI: State trait anxiety inventory
SWLS: Satisfaction with life scale
WFIRS: Weiss functional impairment rating scale
WURS: Wender utah rating scale
Zoo Map (BADS): Zoo map for the behavioral assessment of the dysexecutive syndrome
