# Supplementary material for: Psychoeducational group interventions for adults diagnosed with attention-deficit/ hyperactivity disorder: a scoping review of feasibility, acceptability, and outcome measures
Source: BMC Psychiatry. 2024 Jun 20;24:463. doi: 10.1186/s12888-024-05908-8 (PMC11191191; doi:10.1186/s12888-024-05908-8)
Supplement: Supplementary file 1 — Additional file 1. [file 12888_2024_5908_MOESM1_ESM.docx]

Additional file1. Searching strategy

Article Title: Psychoeducational Group Interventions for Adults Diagnosed with Attention-Deficit/ Hyperactivity Disorder: A Scoping Review of Feasibility, Acceptability, and Outcome Measures

Corresponding author email: <tatiana.skliarova@ntnu.no>

**MEDLINE via EBSCOhost**

| Row | Query |
| --- | --- |
| S1 | (MH "Patient Education as Topic+") |
| S2 | TI patient? N2 educat* OR AB patient? N2 educat* |
| S3 | TI psychoeducat* OR AB psychoeducat* |
| S4 | TI "psycho educat*" OR AB "psycho educat*" |
| S5 | S1 OR S2 OR S3 OR S4 |
| S6 | (MH "Attention Deficit and Disruptive Behavior Disorders+") |
| S7 | (MH "Attention Deficit Disorder with Hyperactivity") |
| S8 | (MH "Hyperkinesis") |
| S9 | TI (attention* or inattent* or impuls* or defian* or opposition* or disruptive* or hyperactive* or "hyper active*" or hyperkine* or "hyper kine*" or "minimal brain" or conduct) N2 (disorder? or syndrome* or behavio?r? or deficit* or deficien* or function*) OR AB (attention* or inattent* or impuls* or defian* or opposition* or disruptive* or hyperactive* or "hyper active*" or hyperkine* or "hyper kine*" or "minimal brain" or conduct) N2 (disorder? or syndrome* or behavio?r? or deficit* or deficien* or function*) |
| S10 | TI ADHD OR AB ADHD |
| S11 | TI ADDH OR AB ADDH |
| S12 | TI ADHS OR AB ADHS |
| S13 | TI "AD HD" OR "AD HD". |
| S14 | TI HKD OR AB HKD |
| S15 | TI ODD OR AB ODD |
| S16 | TI TOC OR AB TOC |
| S17 | TI TDAH OR AB TDAH |
| S18 | S6 OR S7 OR S8 OR S9 OR S10 OR S11 OR S12 OR S13 OR S14 OR S15 OR S16 OR S17 |
| S19 | S5 AND S18 |

**MEDLINE via Ovid**

| # | Searches |
| --- | --- |
| 1 | psychoeducat*.ti,ab,kf. |
| 2 | "psycho educat*".ti,ab,kf. |
| 3 | or/1-2 [Concept #1: Psychoeducation] |
| 4 | "Attention Deficit and Disruptive Behavior Disorders"/ |
| 5 | exp Attention Deficit Disorder with Hyperactivity/ |
| 6 | exp Hyperkinesis/ |
| 7 | ((attention* or inattent* or impuls* or defian* or opposition* or disruptive* or hyperactive* or "hyper active*" or hyperkine* or "hyper kine*" or "minimal brain" or conduct) adj3 (disorder? or syndrome* or behavi?r? or deficit* or deficien* or function*)).ti,ab,kf. |
| 8 | ADHD.ti,ab,kf. |
| 9 | ADDH.ti,ab,kf. |
| 10 | ADHS.ti,ab,kf. |
| 11 | "AD HD".ti,ab,kf. |
| 12 | HKD.ti,ab,kf. |
| 13 | ODD.ti,ab,kf. |
| 14 | TOC.ti,ab,kf. |
| 15 | TDAH.ti,ab,kf. |
| 16 | or/4-15 [Concept #2: Attention Deficit Disorders] |
| 17 | and/3,16 [Concept #1 and #2 combined] |

**Cochrane Library**

ID Search Hits

#1 MeSH descriptor: [Patient Education as Topic] explode all trees

#2 (patient* NEAR/3 educat*):ti,ab,kw (Word variations have been searched)

#3 psychoeducat*:ti,ab,kw

#4 psycho-educat*:ti,ab,kw

#5 #1 OR #2 OR #3 OR #4

#6 MeSH descriptor: [Attention Deficit and Disruptive Behavior Disorders] this term only

#7 MeSH descriptor: [Attention Deficit Disorder with Hyperactivity] explode all trees

#8 ((attention* or inattent* or impuls* or defian* or opposition* or disruptive* or hyperactive* or hyper-active* or hyperkine* or hyper-kine* or minimal-brain or conduct) NEAR/3 (disorder? or syndrome* or behavio?r? or deficit* or deficien* or function*)):ti,ab,kw

#9 ADHD:ti,ab,kw

#10 ADDH:ti,ab,kw

#11 ADHS:ti,ab,kw

#12 AD-HD:ti,ab,kw

#13 HKD:ti,ab,kw

#14 ODD:ti,ab,kw

#15 TOC:ti,ab,kw

#16 TDAH:ti,ab,kw

#17 #6 OR #7 OR #8 OR #9 OR #10 OR #11 OR #12 OR #13 OR #14 OR #15 OR #16

#18 #5 AND #17

**Embase.com**

#1. 'patient education'/exp

#2. (patient$ NEAR/3 educat*):ti,ab,kw

#3. psychoeducat*:ti,ab,kw

#4. 'psycho educat*':ti,ab,kw

#5. #1 OR #2 OR #3 OR #4

#6. 'attention deficit hyperactivity disorder'/exp

#7. ((attention* OR inattent* OR impuls* OR defian* OR opposition* OR disruptive* OR hyperactive* OR 'hyper active*' OR hyperkine* OR 'hyper kine*' OR 'minimal brain' OR conduct) NEAR/3 (disorder? OR syndrome* OR behavio$r$ OR deficit* OR deficien* OR function*)):ti,ab,kw

#8. adhd:ti,ab,kw

#9. addh:ti,ab,kw

#10. adhs:ti,ab,kw

#11. 'ad hd':ti,ab,kw

#12. hkd:ti,ab,kw

#13. odd:ti,ab,kw

#14. tocti,ab,kw

#15. toc:ti,ab,kw

#16. tdah:ti,ab,kw

#17. #6 OR #7 OR #8 OR #9 OR #10 OR #11 OR #12 OR #13 OR #14 OR #15 OR #16

#18. #5 AND #17

**Embase via Ovid**

| **#** | **Searches** |  |  |  |
| --- | --- | --- | --- | --- |
| 1 | exp patient education/ |  |  |  |
| 2 | (patient? adj3 educat*).ti,ab,kw. |  |  |  |
| 3 | psychoeducat*.ti,ab,kw. |  |  |  |
| 4 | "psycho educat*".ti,ab,kw. |  |  |  |
| 5 | or/1-4 [Concept #1: Psychoeducation] |  |  |  |
| 6 | exp attention deficit disorder/ |  |  |  |
| 7 | ((attention* or inattent* or impuls* or defian* or opposition* or disruptive* or hyperactive* or "hyper active*" or hyperkine* or "hyper kine*" or "minimal brain" or conduct) adj3 (disorder? or syndrome* or behavio?r? or deficit* or deficien* or function*)).ti,ab,kw. |  |  |  |
| 8 | ADHD.ti,ab,kw. |  |  |  |
| 9 | ADDH.ti,ab,kw. |  |  |  |
| 10 | ADHS.ti,ab,kw. |  |  |  |
| 11 | "AD HD".ti,ab,kw. |  |  |  |
| 12 | HKD.ti,ab,kw. |  |  |  |
| 13 | ODD.ti,ab,kw. |  |  |  |
| 14 | TOC.ti,ab,kw. |  |  |  |
| 15 | TDAH.ti,ab,kw. |  |  |  |
| 16 | or/6-15 [Concept #2: Attention Deficit Disorders] |  |  |  |
| 17 | and/5,16 [Concept #1 and #2 combined] |  |  |  |

**PsycINFO via Ovid**

| **#** | **Searches** |  |  |  |
| --- | --- | --- | --- | --- |
| 1 | exp Client Education/ |  |  |  |
| 2 | (patient? adj3 educat*).ti,ab,id. |  |  |  |
| 3 | psychoeducat*.ti,ab,id. |  |  |  |
| 4 | "psycho educat*".ti,ab,id. |  |  |  |
| 5 | or/1-4 [Concept #1: Psychoeducation] |  |  |  |
| 6 | exp Attention Deficit Disorder/ |  |  |  |
| 7 | exp Attention Deficit Disorder with Hyperactivity/ |  |  |  |
| 8 | exp Hyperkinesis/ |  |  |  |
| 9 | ((attention* or inattent* or impuls* or defian* or opposition* or disruptive* or hyperactive* or "hyper active*" or hyperkine* or "hyper kine*" or "minimal brain" or conduct) adj3 (disorder? or syndrome* or behavio?r? or deficit* or deficien* or function*)).ti,ab,id. |  |  |  |
| 10 | ADHD.ti,ab,id. |  |  |  |
| 11 | ADDH.ti,ab,id. |  |  |  |
| 12 | ADHS.ti,ab,id. |  |  |  |
| 13 | "AD HD".ti,ab,id. |  |  |  |
| 14 | HKD.ti,ab,id. |  |  |  |
| 15 | ODD.ti,ab,id. |  |  |  |
| 16 | TOC.ti,ab,id. |  |  |  |
| 17 | TDAH.ti,ab,id. |  |  |  |
| 18 | or/6-17 [Concept #2: Attention Deficit Disorders] |  |  |  |
| 19 | and/5,18 [Concept #1 and #2 combined] |  |  |  |

**Web of Science**

| 1 | "patient?" NEAR/3 "educat*" (Topic) |
| --- | --- |
| 2 | "psychoeducat*" (Topic) |
| 3 | "psycho educat*" (Topic) |
| 4 | #3 OR #2 OR #1 |
| 5 | ("attention*" or "inattent*" or "impuls*" or "defian*" or "opposition*" or "disruptive*" or "hyperactive*" or "hyper active*" or "hyperkine*" or "hyper kine*" or "minimal brain" or "conduct") NEAR/2 ("disorder?" or "syndrome*" or "behavio?r?" or "deficit*" or "deficien*" or "function*") (Topic) |
| 6 | "ADHD" (Topic) |
| 7 | "ADDH" (Topic) |
| 8 | "ADHS" (Topic) |
| 9 | "AD HD" (Topic) |
| 10 | "HKD" (Topic) |
| 11 | "ODD" (Topic) |
| 12 | "TOC" (Topic) |
| 13 | "TDAH" (Topic) |
| 14 | #5 OR #6 OR #7 OR #8 OR #9 OR #10 OR #11 OR #12 OR #13 |
| 15 | #4 AND #14 |

**AMED via Ebsconet**

| **#** | **Searches** |  |  |  |
| --- | --- | --- | --- | --- |
| 1 | exp Patient education/ |  |  |  |
| 2 | (patient? adj3 educat*).ti,ab,et. |  |  |  |
| 3 | psychoeducat*.ti,ab,et. |  |  |  |
| 4 | "psycho educat*".ti,ab,et. |  |  |  |
| 5 | or/1-4 [Concept #1: Psychoeducation] |  |  |  |
| 6 | exp Attention deficit disorder with hyperactivity/ |  |  |  |
| 7 | exp Hyperkinesis/ |  |  |  |
| 8 | ((attention* or inattent* or impuls* or defian* or opposition* or disruptive* or hyperactive* or "hyper active*" or hyperkine* or "hyper kine*" or "minimal brain" or conduct) adj3 (disorder? or syndrome* or behavio?r? or deficit* or deficien* or function*)).ti,ab,et. |  |  |  |
| 9 | ADHD.ti,ab,et. |  |  |  |
| 10 | ADDH.ti,ab,et. |  |  |  |
| 11 | ADHS.ti,ab,et. |  |  |  |
| 12 | "AD HD".ti,ab,et. |  |  |  |
| 13 | HKD.ti,ab,et. |  |  |  |
| 14 | ODD.ti,ab,et. |  |  |  |
| 15 | TOC.ti,ab,et. |  |  |  |
| 16 | TDAH.ti,ab,et. |  |  |  |
| 17 | or/6-16 [Concept #2: Attention Deficit Disorders] |  |  |  |
| 18 | and/5,17 [Concept #1 and #2 combined] |  |  |  |

**CINAHL via Ebscohost**

# Query

S1 (MH "Patient Education+")

S2 TI patient? N2 educat* OR AB patient? N2 educat*

S3 TI psychoeducat* OR AB psychoeducat*

S4 TI "psycho educat*" OR AB "psycho educat*"

S5 S1 OR S2 OR S3 OR S4

S6 (MH "Attention Deficit Hyperactivity Disorder")

S7 (MH "Hyperkinesis")

S8 TI ( (attention* or inattent* or impuls* or defian* or opposition* or disruptive* or hyperactive* or "hyper active*" or hyperkine* or "hyper kine*" or "minimal brain" or conduct) N2 (disorder# or syndrome# or behavio#r# or deficit# or deficien# or function*) ) OR AB ( (attention* or inattent* or impuls* or defian* or opposition* or disruptive* or hyperactive* or "hyper active*" or hyperkine* or "hyper kine*" or "minimal brain" or conduct) N2 (disorder# or syndrome# or behavio#r# or deficit# or deficien# or function*) )

S9 TI ADHD OR AB ADHD

S10 TI ADDH OR AB ADDH

S11 TI ADHS OR AB ADHS

S12 TI "AD HD" OR AB "AD HD"

S13 TI HKD OR AB HKD

S14 TI ODD OR AB ODD

S15 TI TOC OR AB TOC

S16 TI TDAH OR AB TDAH

S17 S6 OR S7 OR S8 OR S9 OR S10 OR S11 OR S12 OR S13 OR S14 OR S15 OR S16

S18 S5 AND S17

**ClinicalTrials.gov**

("attention*" OR "inattent*" OR "impuls*" OR "defian*" OR "opposition*" OR "disruptive*" OR "hyperactive*" OR "hyper active*" OR "hyperkine*" OR "hyper kine*" OR "minimal brain") AND ("patient education" OR "psychoeducat*" OR "psyco educat*")
