# Supplementary material for: Psychoeducational group interventions for adults diagnosed with attention-deficit/ hyperactivity disorder: a scoping review of feasibility, acceptability, and outcome measures
Source: BMC Psychiatry. 2024 Jun 20;24:463. doi: 10.1186/s12888-024-05908-8 (PMC11191191; doi:10.1186/s12888-024-05908-8)
Supplement: Supplementary file 2 — Additional file 2. [file 12888_2024_5908_MOESM2_ESM.docx]

Additional file 2. Data extraction quality of reporting

Article Title: Psychoeducational Group Interventions for Adults Diagnosed with Attention-Deficit/ Hyperactivity Disorder: A Scoping Review of Feasibility, Acceptability, and Outcome Measures

Corresponding author email: <tatiana.skliarova@ntnu.no>

Table AF_2 Reporting quality of feasibility and RCT studies

| **Topic** | **Item No*** | | **Checklist item** | | **Vidal**  **et al.,**  **2013** | | **Hirvikoski et al.,**  **2015** | | **Hirvikoski et al.,**  **2017** | **Hoxhaj**  **et al.,**  **2018** | | **In de Braek**  **et al.,**  **2017** | | **Bachmann et al.,**  **2018** | **Hartung**  **et al.,**  **2022** | | **Selaskowski et al.,**  **2022** |
| --- | --- | --- | --- | --- | --- | --- | --- | --- | --- | --- | --- | --- | --- | --- | --- | --- | --- |
| **Title and**  **abstract** | 1a | | Identification as a pilot or feasibility randomised trial in the title | | Yes,  p.894 | | Yes,  p.89 | | Yes,  p.141 | Yes,  p.321 | | No | | Yes,  p.47 | No | | Yes,  p.1 |
|  | 1b | | Structured summary of pilot trial design, methods, results, and conclusions (for specific guidance see CONSORT abstract extension for pilot trials) | | No | | No | | Yes,  p.141 | Yes,  p.321 | | Yes,  p.1130 | | Yes,  p. 47 | Yes,  p.411 | | No |
| Introduction | | | | |  | |  | |  |  | |  | |  |  | |  |
| Background  and objectives | 2a | | Scientific background and explanation of rationale for future definitive trial, and reasons for randomised pilot trial | | Yes,  p.894-895 | | Yes,  p.89-90 | | Yes,  p.141-142 | Yes  p.321-322 | | Yes,  p.1130-1131 | | Yes,  p.47 - 48 | Yes,  p. 413-414 | | Yes,  p.1-2 |
|  | 2b | | Specific objectives or research questions for pilot trial | | Yes,  p.895 | | Yes,  p.90 | | Yes,  p.142 | Yes,  p.322 | | Yes,  p.1131 | | Yes,  p.48 | Yes,  p.414 | | Yes,  p.2 |
| Methods | | | | |  | |  | |  |  | |  | |  |  | |  |
| Trial design | 3a | | Description of pilot trial design (such as parallel, factorial) including allocation ratio | | Yes,  p.895 | | Yes,  p.90-92 | | Yes,  p.142-143 | Yes  p.322-323 | | No | | Yes,  p.49 | No | | Yes,  p.3 |
|  | 3b | | Important changes to methods after pilot trial commencement (such as eligibility criteria), with reasons | | No | | No | | No | No | | Yes,  p.1131 | | No | No | | No |
| Participants | 4a | | Eligibility criteria for participants | | Yes,  p.895 | | Yes,  p.91 | | Yes,  p.142 | Yes,  p.322 | | Yes,  p.1131 | | Yes,  p.48 | Yes,  p.414-415 | | Yes,  p.2 |
|  | 4b | | Settings and locations where the data were collected | | Yes,  p. 895 | | Yes,  p.90 | | Yes,  p.142 | Yes,  p.322 | | Yes,  p.1131 | | Yes,  p.48 | Yes,  p.414 | | Yes,  p.2 |
|  | 4c | | How participants were identified and consented | | Yes,  p.897 | | Yes,  p.91 | | Yes,  p.142 | Yes,  p.322 | | Yes,  p.1131 | | Yes,  p.48 | Yes,  p.414-415 | | Yes,  p.2 |
| Interventions | 5 | | The interventions for each group with sufficient details to allow replication, including how and when they were actually administered | | Yes,  p.895-896 | | Yes,  p.91-92 | | Yes,  p.144-145 | Yes,  p.323 | | Yes,  p.1132-1133 | | Yes,  p.49 | Yes,  p.415 | | Yes,  p.3 |
| Outcomes | 6a | | Completely defined prespecified assessments or measurements to address each pilot trial objective specified in 2b, including how and when they were assessed | | Yes,  p.896 | | Yes,  p.92-93 | | Yes,  p.144-145 | Yes,  p.323 | | Yes,  p.1133-1134 | | Yes,  p.48-49 | Yes,  p.416-417 | | Yes,  p.4 |
|  | 6b | | Any changes to pilot trial assessments or measurements after the pilot trial commenced, with reasons | | No | | No | | No | No | | No | | No | No | | No |
|  | 6c | | If applicable, prespecified criteria used to judge whether, or how, to proceed with future definitive trial | | No | | No | | No | No | | No | | No | No | | No |
| Sample size | 7a | | Rationale for numbers in the pilot trial | | No | | No | | No | Yes,  p.324 | | No | | No | No | | No |
|  | 7b | | When applicable, explanation of any interim analyses and stopping guidelines | | No | | No | | No | No | | No | | No | No | | No |
| Randomisation |  | |  | |  | |  | |  |  | |  | |  |  | |  |
| Sequence  generation | 8a | | Method used to generate the random allocation sequence | | Yes,  p.896 | | No | | Yes,  p.144 | Yes,  p.324 | | No | | Yes,  p.49 | No | | No |
|  | 8b | | Type of randomisation(s); details of any restriction (such as blocking and block size) | | No | | No | | Yes,  p.144 | No | | No | | No | No | | Yes,  p.3 |
| Allocation  concealment  mechanism | 9 | | Mechanism used to implement the random allocation sequence (such as sequentially numbered containers), describing any steps taken to conceal the sequence until interventions were assigned | | No | | No | | No | No | | No | | No | No | | No |
| Implementation | 10 | | Who generated the random allocation sequence, who enrolled participants, and who assigned participants to interventions | | No | | No | | Yes,  p.144 | Yes,  p.322-324 | | No | | Yes,  p.48-49 | Yes,  p.414-415 | | No |
| Blinding | 11a | | If done, who was blinded after assignment to interventions (for example, participants, care providers, those assessing outcomes) and how | | No | | No | | Yes,  p.144 | No | | Yes,  p.1133 | | No | No | | No |
|  | 11b | | If relevant, description of the similarity of interventions | | No | | No | | No | No | | No | | No | No | | No |
| Statistical methods | 12 | | Methods used to address each pilot trial objective whether qualitative or quantitative | | Yes,  p.897 | | Yes,  p.93 | | Yes,  p.143-144 | Yes,  p.324 - 331 | | Yes,  p.1133-1134 | | Yes,  p.49-50 | Yes,  p.418-419 | | Yes,  p.4 |
| Results | |  | |  | |  | |  | | |  | |  | | |  | |
| Participant  flow (a diagram is strongly recommended) | 13a | | For each group, the numbers of participants who were approached and/or assessed for eligibility, randomly assigned, received intended treatment, and were assessed for each objective | | Yes,  p.896 | | Yes,  p.94-95 | | Yes,  p.145 | Yes,  p.325 | | No | | Yes,  p.49 | Yes,  p.415 | | Yes,  p.2 |
|  | 13b | | For each group, losses and exclusions after randomisation, together with reasons | | Yes,  p.896 | | Yes,  p.91 | | Yes,  p.145 | Yes,  p.325 | | No | | Yes,  p.49 | Yes,  p.415 | | Yes,  p.2 |
| Recruitment | 14a | | Dates defining the periods of recruitment and follow-up | | No | | No | |  | Yes,  p.323 | | Yes,  p.1131 | | No | No | | Yes,  p.4 |
|  | 14b | | Why the pilot trial ended or was stopped | | No | | No | | No | No | | No | | No | No | | No |
| Baseline data | 15 | | A table showing baseline demographic and clinical characteristics for each group | | Yes,  p.897 | | Yes,  p.94 | | Yes,  p.146 | Yes,  p.326-331 | | Yes,  p.1132 | | Yes,  p.51 | No | | Yes,  p.5 |
| Numbers analysed | 16 | | For each objective, number of participants (denominator) included in each analysis. If relevant, these numbers  should be by randomised group | | No | | No | | No | No | | No | | No | No | | Yes,  p.5 |
| Outcomes and estimation | 17 | | For each objective, results including expressions of uncertainty (such as 95% confidence interval) for any  estimates. If relevant, these results should be by randomised group | | Yes,  p.897-898 | | No | | Yes,  p.148 | No | | Yes,  p.1134 | | Yes,  p.52 | Yes,  p.419 | | Yes,  p.4-5 |
| Ancillary analyses | 18 | | Results of any other analyses performed that could be used to inform the future definitive trial | | No | | No | | No | No | | No | | No | No | | No |
| Harms | 19 | | Harms or unintended effects in each group (for specific guidance see CONSORT for harms) | | No | | No | | No | No | | No | | No | No | | No |
|  | 19a | | If relevant, other important unintended consequences | | No | | No | | No | No | | No | | No | No | | No |
| Discussion | |  | |  | |  | |  | | |  | |  | | |  | |
| Limitations | 20 | | Pilot trial limitations, addressing sources of potential bias and remaining uncertainty about feasibility | | Yes,  p.899 | | Yes,  p.97 | | Yes,  p.150 | Yes,  p.333 | | Yes,  p.1136 | | Yes,  p.54 | Yes,  p.421 | | No |
| Generalisability | 21 | | Generalisability (applicability) of pilot trial methods and findings to future definitive trial and other studies | | Yes,  p.899 | | No | | No | Yes,  p.333 | | No | | Yes,  p.54 | Yes,  p.421 | | Yes,  p.5-7 |
| Interpretation | 22 | | Interpretation consistent with pilot trial objectives and findings, balancing potential benefits and harms, and  considering other relevant evidence | | Yes,  p.898-899 | | Yes,  p.97 | | Yes,  p.149-150 | Yes,  p.331-332 | | Yes,  p.1135-1136 | | Yes  p.52-54 | Yes  p.420-421 | | Yes,  p. 5-7 |
|  | 22a | | Implications for progression from pilot to future definitive trial, including any proposed amendments | | Yes,  p.899 | | Yes,  p.97 | | No | No | | No | | No | No | | Yes,  p.7 |
| Other information | | | | |  | |  | |  |  | |  | |  |  | |  |
| Registration | 23 | | Registration number for pilot trial and name of trial registry | | No | | Yes,  p.98 | | Yes,  p.150 | No | | No | | Yes,  p.48,55 | No | | Yes,  p.3 |
| Protocol | 24 | | Where the pilot trial protocol can be accessed, if available | | No | | No | | No | No | | No | | No | No | | No |
| Funding | 25 | | Sources of funding and other support (such as supply of drugs), role of funders | | No | | Yes,  p.98 | | Yes,  p.150 | No | | Yes,  p.1136 | | Yes  p.55 | No | | Yes,  p.7 |
|  | 26 | | Ethical approval or approval by research review committee, confirmed with reference number | | Yes,  p.897 | | Yes,  p.98 | | Yes,  p.142 | Yes,  p.323 | | No | | Yes,  p.48 | No | | Yes,  p.2 |

**Note:** *The checklist items of data Consolidated Standards of Reporting of Trial was used to evaluate the quality of reporting of feasibility and RCT-studies
