# Supplementary material for: Psychoeducational group interventions for adults diagnosed with attention-deficit/ hyperactivity disorder: a scoping review of feasibility, acceptability, and outcome measures
Source: BMC Psychiatry. 2024 Jun 20;24:463. doi: 10.1186/s12888-024-05908-8 (PMC11191191; doi:10.1186/s12888-024-05908-8)
Supplement: Supplementary file 4 — Additional file 4. [file 12888_2024_5908_MOESM4_ESM.docx]

**Additional file 4:** Excluded articles and reasons of exclusion

**Article Title:** Psychoeducational Group Interventions for Adults Diagnosed with Attention-Deficit/ Hyperactivity Disorder: A Scoping Review of Feasibility, Acceptability, and Outcome Measures

**Corresponding Author email:** [tatiana.skliarova@ntnu.no](mailto:tatiana.skliarova@ntnu.no)

**Table AF_4.** Excluded articles after eligibility through full-text study and reasons for the exclusion

| **Exclusion criteria** | **First author, year of publication [reference]** | **Reasons** |
| --- | --- | --- |
| No psychoeducational intervention | Cellucci, T., 2007 [1] | A psycho-social evaluation and assessment intervention, based on the assessment results, personalized recommendations were provided to each student |
|  | De Oliveira, C.T., 2018 [2] | An online educational booklet intervention and participants accessed the booklet through an online platform |
|  | Hanssen, K.T., 2023 [3] | A study focused on Goal-based Management Training and metacognitive skills |
|  | Jang, S., 2021 [4] | A mobile app-based chatbot intervention focusing on cognitive behavioral therapy technics and skills |
|  | Prevatt, F., 2017 [5] | A study about individualized ADHD coaching |
|  | Safren, S.A., 2010 [6] | A study comparing individual sessions of cognitive behavioral therapy and relaxation with educational support |
|  | Salomone, S., 2012 [7] | An individual self-alert training, with skin-conductance biofeedback program |
|  | Sehlin, H., 2020 [8] | An internet-based support and coaching intervention, with two face-to-face clinic visits with coaches |
|  | Vasko, J.M., 2019 [9] | A mixed intervention, combining different approaches including motivational interview, psychoeducation, behavioral activation, or supportive counselling |
|  | Wiggins, D., 1999 [10] | A group counselling intervention aimed to enhance organizational skills, self-awareness, and adaptive behaviors among adults with ADHD |
| Other reasons | Bemporad, J., 1996[11] | Not Psychoeducational Intervention. Theoretical article about psychotherapy of adults with ADHD |
|  | Hirvikoski, T., 2015[12] | Conference paper |
|  | Pheh, K.S., 2021 [13] | Protocol study |
|  | Selaskovski, S., 2023 [14] | An interactive chatbot and self-guided intervention |

**References**

1. Cellucci T, Remsperger P, McGlade E: **Psycho-educational evaluations for university students in one clinic**. *Psychological Reports* 2007, **101**(2):501-511.

2. de Oliveira CT, Teixeira MAP, Dias ACG: **Effectiveness of a psychoeducational booklet on ADHD in college students**. *Psicologia: Teoria e Pratica* 2018, **20**(2):281-292.

3. Hanssen KT, Brevik EJ, Småstuen MC, Stubberud J: **Improvement of anxiety in ADHD following goal-focused cognitive remediation: a randomized controlled trial**. *Front Psychol* 2023, **14**:1212502.

4. Jang S, Kim JJ, Kim SJ, Hong J, Kim S, Kim E: **Mobile app-based chatbot to deliver cognitive behavioral therapy and psychoeducation for adults with attention deficit: A development and feasibility/usability study**. *International Journal of Medical Informatics* 2021, **150**:104440.

5. Prevatt F, Smith SM, Diers S, Marshall D, Coleman J, Valler E, Miller N: **ADHD Coaching With College Students: Exploring the Processes Involved in Motivation and Goal Completion**. *Journal of College Student Psychotherapy* 2017, **31**(2):93-111.

6. Safren SA, Sprich S, Mimiaga MJ, Surman C, Knouse L, Groves M, Otto MW: **Cognitive behavioral therapy vs relaxation with educational support for medication-treated adults with ADHD and persistent symptoms: a randomized controlled trial**. *Jama* 2010, **304**(8):875-880.

7. Salomone S, Shanahan JM, Bramham J, O'Connell RG, Robertson IH: **A biofeedback-based programme to improve attention and impulsivity in adults with ADHD**. *The Irish Journal of Psychology* 2012, **33**(2-3):86-93.

8. Sehlin H, Ahlstrom BH, Bertilsson I, Andersson G, Wentz E: **Internet-based support and coaching with complementary clinic visits for young people with attention-deficit/hyperactivity disorder and autism: Controlled feasibility study**. *Journal of Medical Internet Research* 2020, **22**(12).

9. Vasko JM, Meinzer MC, Murphy JG, Oddo LE, McCauley KL, Rooney ME, Lejuez CW, Chronis-Tuscano A: **Brief Intervention to Reduce Problem Drinking in College Students With ADHD**. *Cognitive and Behavioral Practice* 2019, **26(3)**:506-521.

10. Wiggins D, Singh K, Getz HG, Hutchins DE: **Effects of brief group intervention for adults with attention deficit/hyperactivity disorder**. *Journal of Mental Health Counseling* 1999, **21**(1):82-92.

11. Bemporad J, Zambenedetti M: **Psychotherapy of adults with attention-deficit disorder**. *Journal of Psychotherapy Practice & Research* 1996, **5**(3):228-237.

12. Hirvikoski T, Waaler E, Lindström T, Bölte S, Jokinen J: **Psychoeducational groups for adults with ADHD and their significant others (PEGASUS): an open clinical feasibility trial**. *ADHD attention deficit and hyperactivity disorders* 2015, **7**:S94‐.

13. Pheh KS, Tan KA, Ibrahim N, Sidik SM: **Effectiveness of online mindfulness-based intervention (Imbi) on inattention, hyperactivity–impulsivity, and executive functioning in college emerging adults with attention-deficit/hyperactivity disorder: A study protocol**. *International Journal of Environmental Research and Public Health* 2021, **18**(3):1‐12.

14. Selaskowski B, Reiland M, Schulze M, Aslan B, Kannen K, Wiebe A, Wallbaum T, Boll S, Lux S, Philipsen A *et al*: **Chatbot-supported psychoeducation in adult attention-deficit hyperactivity disorder: randomised controlled trial**. *BJPsych Open* 2023, **9**(6):e192.
