# Supplementary material for: Psychoeducational group interventions for adults diagnosed with attention-deficit/ hyperactivity disorder: a scoping review of feasibility, acceptability, and outcome measures
Source: BMC Psychiatry. 2024 Jun 20;24:463. doi: 10.1186/s12888-024-05908-8 (PMC11191191; doi:10.1186/s12888-024-05908-8)
Supplement: Supplementary file 5 — Additional file 5. [file 12888_2024_5908_MOESM5_ESM.docx]

**Psychoeducational Group Interventions for Adults Diagnosed with ADHD: A Scoping Review Protocol**

**ABSTRACT**

For adult patients, psychoeducational group interventions are increasingly being used as an alternative or supplement to pharmacological treatment. Notably lacking, however, is a scoping review specifically focusing on the feasibility and acceptability of such interventions. Therefore, the goal of the present protocol is to describe and conduct a scoping review to map out the existing literature on psychoeducation group interventions for adults diagnosed with attention-deficit/hyperactivity disorder (ADHD). The scoping review aims to comprehensively examine the feasibility indicators, acceptability, and outcomes used in such interventions in the context of adult ADHD. By doing this, we aim to address an important knowledge gap, providing valuable insights for clinicians involved in the group treatment of adults diagnosed with ADHD. The results could be valuable for researchers and help to guide the future research, development and implementation of interventions in clinical settings.

The framework developed by the Joanna Briggs Institute (JBI) will be used as a guide in this scoping process. A search strategy with broad searching terms will be conducted in Ovid MEDLINE, Embase, PsycINFO, Web of Science, Cochrane CENTRAL, AMED, and ClinicalTrials.gov. The databases were searched for trials in May 2022 and January 2024. Two or more reviewers will be included in every review step. The review will use the Preferred Reporting Items for Systematic reviews and Meta-Analyses extension for Scoping Reviews (PRISMA-ScR) checklist, as well as TIDieR and CONSORT, when reporting its results.

**Keywords:** Attention-deficit/hyperactivity disorder, ADHD, feasibility, patient education, psychoeducational intervention, scoping review, treatment outcomes, protocol, scoping review

**STRENGTHS AND LIMITATIONS**

- To ensure systematic and transparent methods that are aligned with the most current guidance for scoping reviews, this protocol is guided by CONSORT, TIDieR, and the PRISMA Scoping Reviews guidelines.
- The research questions, search strategy, and data-charting methods are informed by an interdisciplinary team of clinicians, a user representative, and scoping-review methodologists.
- This scoping review address a gap in the current literature on psychoeducational group interventions.
- This review’s findings will add to the evidence of the characteristics of outcomes used when evaluating psychoeducational group interventions; this may inform and guide future research.
- This review will be limited to publications in English.

**BACKGROUND**

Attention-deficit/hyperactivity disorder (ADHD) is a neurodevelopmental disorder characterized by symptoms of inattention, impulsivity, and hyperactivity ([Zalsman & Shilton, 2016](#_ENREF_28)). It typically begins in childhood, and, while about 2.5% of adults experience persistent ADHD, approximately 6.7% of adults worldwide exhibit symptomatic ADHD ([Song et al., 2021](#_ENREF_25)). This condition is known to cause psychosocial impairments, including lower rates of completing higher education, difficulties in the workplace, higher risk of accidents ([Biederman et al., 2006](#_ENREF_2); [Gjervan et al., 2012](#_ENREF_6)), and drug and alcohol abuse ([Biederman et al., 2020](#_ENREF_3); [Torgersen et al., 2006](#_ENREF_26)). Furthermore, ADHD frequently co-occurs with anxiety and depression ([Katzman et al., 2017](#_ENREF_10)). Studies also suggest that individuals with ADHD often have lower self-management skills ([Newark et al., 2016](#_ENREF_14); [Newark & Stieglitz, 2010](#_ENREF_15)).

In the context of ADHD, having knowledge and self-management skills holds significant importance for adults receiving treatment in mental-health services. Specifically, higher levels of self-efficacy in individuals with ADHD can lead to a greater belief in their own ability to cope effectively with the challenges of daily life (Newark & Stieglitz, 2010). One promising approach to improving knowledge and self-management for people with ADHD is psychoeducation. Promising evidence supports the effectiveness of psychoeducational interventions ([Scholz et al., 2020](#_ENREF_21)) and self-management programs ([Leitan et al., 2015](#_ENREF_13)) to enhance the active involvement of patients in their own healthcare. Systematic reviews examining strategies to promote patient engagement have consistently demonstrated the benefits of educational interventions for adults with various mental-health conditions ([Greene et al., 2016](#_ENREF_7); [Keen et al., 2021](#_ENREF_11); [Leitan et al., 2015](#_ENREF_13); [Siantz & Aranda, 2014](#_ENREF_23)). Specifically, these interventions have proven effective in improving mental-health knowledge and attitudes, enhancing patient activation, and addressing barriers to treatment ([Beentjes et al., 2020](#_ENREF_1); [Kelly et al., 2014](#_ENREF_12); [Leitan et al., 2015](#_ENREF_13); [Siantz & Aranda, 2014](#_ENREF_23)). Notably lacking, however, is a scoping review specifically focusing on the feasibility and acceptability of such psychoeducational group interventions for adults diagnosed with ADHD, even though recent studies have shown that adults with ADHD need and want to learn about different aspects of their disorder ([Seery et al., 2022](#_ENREF_22); [Solberg et al., 2019](#_ENREF_24)); ([Seery et al., 2022](#_ENREF_22); [Solberg et al., 2019](#_ENREF_24)). For patients with ADHD, psychoeducational programs help them to acquire knowledge and understand their disorder and the impairments it entails ([Scholz et al., 2020](#_ENREF_21)).

A scoping review conducted in 2018 aiming to identify how researchers characterize the concept of psychoeducation ([Oliveira & Dias, 2018](#_ENREF_17)) included six publications on psychoeducation for adults with ADHD for full review out of 17 total studies included. The only review specifically on psychoeducation and adult ADHD (a rapid review published in 2016) included three studies for full review ([Hafstad & Leiknes, 2016](#_ENREF_8)). As such, while previous research has explored various aspects of psychoeducation for ADHD, such as its definition and approaches used, there remains a limited synthesis of the parameters used to measure patient-reported outcomes and experiences related to psychoeducational group interventions tailored to adults. Furthermore, there has been limited focus on user involvement, either in the development and delivery of interventions, despite the acknowledgement of its importance ([Fullen et al., 2020](#_ENREF_5); [Nimmo-Smith et al., 2020](#_ENREF_16)), or in conducting scoping reviews ([Pollock et al., 2022](#_ENREF_19))**.** In addition, no existing reviews have explicitly investigated the involvement of people close to the patient, and there remains a limited synthesis of the outcome measures. Consequently, this is a study protocol for a scoping review that aims to comprehensively examine the feasibility, acceptability, and outcomes used in such interventions in the context of adult ADHD.

**METHODS**

Scoping reviews are a common approach to evidence synthesis for researchers, clinicians, and policymakers across a variety of fields. The conduct of this protocol was guided by the JBI methodology for scoping reviews ([Peters et al., 2020](#_ENREF_18)), and the proposed scoping review will be conducted and reported in accordance with the Preferred Reporting Items for Systematic Review and Meta-Analysis Protocols (PRISMA-P; ([Tricco et al., 2018](#_ENREF_27)), the Transparent Reporting of Interventions in Educational Research (TIDieR) reporting guidelines, and the Consolidated Standards of Reporting Trials (CONSORT) guidelines ([Sandra et al., 2016](#_ENREF_20)). These reporting standards will contribute to the transparency and rigor of our scoping review. Our review process will be guided by our research questions:

1. What domains or outcomes are included to examine the feasibility of the interventions?
2. What domains are included to examine acceptability?
3. What outcomes and assessment approaches are used in such interventions in the context of adult ADHD?

These questions aim to map the available evidence on psychoeducational group interventions for adults diagnosed with ADHD, emphasizing their feasibility, acceptability, and outcome measures.

To comprehensively gather relevant literature, one of the authors (SP) developed a rigorous search strategy in 2022, which involved searching seven electronic databases for relevant studies published up to our predetermined cutoff date. Our search terms were designed to capture a wide range of articles related to psychoeducational interventions for adults with ADHD.

We aim to employ a systematic approach to study selection, carefully screening articles based on predefined inclusion and exclusion criteria. Eligible studies must focus on psychoeducational group interventions for adults with ADHD and report on aspects of their feasibility, acceptability, and outcome measures.

To synthesize the data from the selected studies, two authors will independently extract all the key information related to the studies’ characteristics, participant demographics, and intervention details. Two authors will independently extract the reported outcomes. This data-charting process, which will use predefined tables, will allow us to extract, organize, and categorize the relevant information. Following the data charting, we will conduct a narrative synthesis of the included studies by summarizing the findings. In addition, throughout the review process, we will engage in consultations with one user representative from a user-led ADHD organization. These consultations will help ensure the accuracy and comprehensiveness of our review findings.

**Searches, eligibility criteria study selection, and data extraction**

The initial searches will be conducted in several databases: Ovid MEDLINE, Embase, PsycINFO, Web of Science, Cochrane CENTRAL, AMED, and ClinicalTrials.gov. The search strategy presented in Table 1 was developed in Ovid MEDLINE and will be adapted to the other databases.

**Table 1**

**Search strategies**

| **Number** | **Searches** |
| --- | --- |
| 1 | exp Patient Education as Topic/ |
| 2 | (patient? adj3 educat*).ti,ab,kf. |
| 3 | psychoeducat*.ti,ab,kf. |
| 4 | "psycho educat*".ti,ab,kf. |
| 5 | or/1-4 [Concept #1: Psychoeducation] |
| 6 | "Attention Deficit and Disruptive Behavior Disorders"/ |
| 7 | exp Attention Deficit Disorder with Hyperactivity/ |
| 8 | exp Hyperkinesis/ ((attention* or inattent* or impuls* or defian* or opposition* or disruptive* or hyperactive* or "hyper active*" or hyperkine* or |
| 9 | "hyper kine*" or "minimal brain" or conduct) adj3 (disorder? or syndrome* or behavio?r? or deficit* or deficien* or function*)).ti,ab,kf. |
| 10 | ADHD.ti,ab,kf. |
| 11 | ADDH.ti,ab,kf. |
| 12 | ADHS.ti,ab,kf. |
| 13 | "AD HD".ti,ab,kf. |
| 14 | HKD.ti,ab,kf. |
| 15 | ODD.ti,ab,kf. |
| 16 | TOC.ti,ab,kf. |
| 17 | TDAH.ti,ab,kf. |
| 18 | or/6-17 [Concept #2: Attention Deficit Disorders] |
| 19 | and/5,18 [Concept #1 and #2 combined] |

The search strategies will aim to locate published studies, and searchers will combine thesaurus and free-text terms related to psychoeducational group interventions. The reference lists of all included sources of evidence will be screened for additional documents. Following the search, all identified citations will be collated into Endnote v.X9. Preliminary results were imported into Endnote 20 reference database software in 2022.

The eligibility criteria will include all peer-reviewed articles that report on findings from a psychoeducational group program. Eligible studies could be pilot studies, clinical studies, feasibility studies, or randomized controlled studies. Studies will be included if they evaluate a psychoeducational group program alone, in comparison to another treatment, or as a control group. The authors will screen the titles and abstracts in duplicate and include all articles that use the terms “ADHD” or “hyperkinetic disorder” and “psychoeducation” or “patient education,” in addition to including only studies with “adults” or “adulthood” in their titles or abstracts.

As *psychoeducational intervention* is a broad term and can be defined as any intervention providing the patient and/or their caregivers with didactic information about the disorder and its treatments to help the patient cope with the problems associated with the disorder ([Ekhtiari et al., 2017](#_ENREF_4)), the definition used in this scoping review is a broad and pragmatic one, to ensure a wide inclusion of studies.

Intervention characteristics will be extracted per the Template for Intervention Description and Replication (TIDieR) checklist (Table 2; ([Hoffmann et al., 2016](#_ENREF_9)).

**Table 2**

**Table for data extraction regarding the intervention using the TIDieR checklist**

| **TIDieR item** | **Brief description** |
| --- | --- |
| Brief name | Brief name of intervention: the name or phrase that describes the intervention |
| Why | Rationale, theory or goal of the intervention |
| What | Physical or informational materials: provided to participants or used in intervention delivery or in training intervention providers |
|  | Procedures, activities and processes: including enabling or support activities |
| Who provided | Intervention provider: including expertise, background and specific training given |
| How | Mode of delivery, for example, face-to-face, internet, telephone and whether provided individually or in a group |
| Where | Mode of delivery, for example, face-to-face, internet, telephone and whether provided individually or in a group |
| When and how much | Timing, duration and Intensity: number of times the intervention was delivered and over what period of time including the number of sessions, their schedule and their duration, intensity or dose |
| Tailoring | Tailoring of Intervention: if planned to be personalised, titrated or adapted, then describe what, why, when and how |
| Modification | Modification of intervention: if modified during the course of the study, describe the changes (what, why, when and how) |
| How well | Planned: if intervention adherence or fidelity was assessed, describe how and by whom, and if any strategies were used to maintain or improve fidelity |
|  | Actual: if intervention adherence or fidelity was assessed, describe the extent to which the intervention was delivered as planned |

The screening of abstracts and titles for inclusion and the subsequent full-text evaluation to evaluate their eligibility for inclusion will be performed by two independent reviewers. After each stage, the reviewers will compare results and discuss potential discrepancies. In cases of disagreement after discussion, a senior member of the team will be consulted. If the title or abstract explicitly states that the participant in the study with ADHD or hyperkinetic disorder is under 18 years old, the study will be excluded. “Psychoeducation” or its related terms must be mentioned in the title or abstract for inclusion. For example, an article titled “Non-Medical Approaches to Treating Adult ADHD” would be included for further investigation even though psychoeducation is not mentioned in the title and might not be mentioned in the abstract. The reasons for excluding full-text articles that do not meet the inclusion criteria will be recorded and reported in a supplementary document in the scoping review. In the full-text review, only psychoeducational group interventions will be eligible for final analysis. Also, at this stage the reference list of relevant review articles will be examined for additional studies, which will be evaluated by the inclusion process already mentioned. Studies will be excluded if they have any of the following characteristics:

1. Studies that do not include adults diagnosed with ADHD;

2. Studies that do not address the main research question or focus on non-psychoeducational interventions;

3. Studies where full-text articles could not be obtained;

4. Non-peer-reviewed articles (e.g., commentaries, opinions, conference abstracts, protocols, theoretical articles, letters);

5. Studies with duplicates or multiple reports of the same data (only the most complete studies with relevant information will be included).

The purpose of extracting the evidence is to detail the results of the scoping review clearly and comprehensibly. The first author (TS), in collaboration with co-authors, will independently extract the data using a pre-defined data-charting form (Tables 3–7). It is expected that the data extracted will include study characteristics, such as the aim, location, methods, and design/type of study, as well as participant characteristics and findings. Psychoeducational interventions have been developed along the mental-health continuum ranging from prevention to treatment, and this scoping review will consider all studies that include at least one outcome measure.

**Table 3**

**Table for data extraction regarding study characteristics**

| **Study ID** |  |
| --- | --- |
| Country, setting |  |
| Design of the study |  |
| N of participants |  |
| Reporting guidelines |  |
| Sample size calculation |  |
| Intervention, description |  |
| Psychoeducation, number of sessions |  |

Conceptual definitions of feasibility indicators and acceptability within the literature vary considerably. Consequently, we anticipate that there will be inconsistencies in how feasibility and acceptability are reported. To allow for an in-depth discussion about the feasibility and acceptability of the study methods and interventions, all measures of feasibility and acceptability will be collected using pre-defined tables with the option of written comments.

**Table 4**

**Table for data extraction regarding the feasibility characteristics of the studies**

Of note, in cases where information is not explicitly reported, it will be marked (using symbol -). This will be equal to not reported.

| **Characteristics of the studies**  Variables |
| --- |
| Dates of recruitment (YYYY.MM) |
| Eligibility rate^1^ |
| Recruitment rate^2^ |
| Attendance to the sessions |
| Retention rate at post intervention |
| Retention rate at follow-up |
| Drop-out rate (*n* and/or %). |
| Evidence of reliability and validity of PROM and PREM in the target population^3^ |
| Resources used |
| Risk assessment |
| Key stakeholders included |
| Statements regarding adaptation for the population |
| Implementation |
| Potential barriers |
| Fidelity |
| Other issues reported |

**^1^** Refers to eligibility rate, and can be defined as the number of participants eligible divided by n of participants screened (*n* or %).

**^2^** Refers to recruitment rate, and is defined as the numbers of participants accepting the invitation, divided by n of participants invited.

**^3^** Refers to reported evidence of reliability, validity of measures in the target population; or there is evidence of psychiatric properties in the target population.

In addition to the acceptability indicators, such as measuring participants’ satisfaction ratings/scores and stakeholders’ acceptance, other quantitative or qualitative data related to feasibility and acceptability of the interventions will be collected to get an overview of tools and methods used for measuring acceptability and areas of concern (Table 5).

**Table 5**

**Table for data extraction regarding acceptability characteristics of the studies**

| **Study ID** |  |
| --- | --- |
| Tools and methods for measuring acceptability |  |
| Stakeholder acceptance |  |
| Consider the emotional and psychological impact of the intervention |  |
| Ethical considerations |  |
| Acceptability or/and patient satisfaction with the group intervention |  |
| Areas of concern |  |
| Contextual factors affecting acceptability |  |

Other measures of feasibility and acceptability will be targeted, including items from the CONSORT 2010 statement (Table 6), to provide an overview of the different approaches used.

**Table 6**

**Table for data extraction quality of reporting studies**

Checklist items* of data to evaluate the quality of reporting of feasibility and RCT-studies

| **Variables** | **Item No*** | **Checklist item** |
| --- | --- | --- |
| Title and  abstract | 1a | Identification as a pilot or feasibility randomised trial in the title |
|  | 1b | Structured summary of pilot trial design, methods, results, and conclusions (for specific guidance see CONSORT abstract extension for pilot trials) |
| Background  and objectives | 2a | Scientific background and explanation of rationale for future definitive trial, and reasons for randomised pilot trial |
|  | 2b | Specific objectives or research questions for pilot trial |
| Methods  Trial design | 3a | Description of pilot trial design (such as parallel, factorial) including allocation ratio |
|  | 3b | Important changes to methods after pilot trial commencement (such as eligibility criteria), with reasons |
| Participants | 4a | Eligibility criteria for participants |
|  | 4b | Settings and locations where the data were collected |
|  | 4c | How participants were identified and consented |
| Interventions | 5 | The interventions for each group with sufficient details to allow replication, including how and when they were actually administered |
| Outcomes | 6a | Completely defined prespecified assessments or measurements to address each pilot trial objective specified in 2b, including how and when they were assessed |
|  | 6b | Any changes to pilot trial assessments or measurements after the pilot trial commenced, with reasons |
|  | 6c | If applicable, prespecified criteria used to judge whether, or how, to proceed with future definitive trial |
| Sample size | 7a | Rationale for numbers in the pilot trial |
|  | 7b | When applicable, explanation of any interim analyses and stopping guidelines |
| Randomisation | 8a | Method used to generate the random allocation sequence |
|  | 8b | Type of randomisation(s); details of any restriction (such as blocking and block size) |
| Allocation  concealment  mechanism | 9 | Mechanism used to implement the random allocation sequence (such as sequentially numbered containers), describing any steps taken to conceal the sequence until interventions were assigned |
| Implementation | 10 | Who generated the random allocation sequence, who enrolled participants, and who assigned participants to interventions |
| Blinding | 11a | If done, who was blinded after assignment to interventions (for example, participants, care providers, those assessing outcomes) and how |
|  | 11b | If relevant, description of the similarity of interventions |
| Statistical methods | 12 | Methods used to address each pilot trial objective whether qualitative or quantitative |
| Results |  |  |
| Participant  flow | 13a | For each group, the numbers of participants who were approached and/or assessed for eligibility, randomly assigned, received intended treatment, and were assessed for each objective |
|  | 13b | For each group, losses and exclusions after randomisation, together with reasons |
| Recruitment | 14a | Dates defining the periods of recruitment and follow-up |
|  | 14b | Why the pilot trial ended or was stopped |
| Baseline data | 15 | A table showing baseline demographic and clinical characteristics for each group |
| Numbers analysed | 16 | For each objective, number of participants (denominator) included in each analysis. If relevant, these numbers should be by randomised group |
| Outcomes and estimation | 17 | For each objective, results including expressions of uncertainty (such as 95% confidence interval) for any estimates. If relevant, these results should be by randomised group |
| Ancillary analyses | 18 | Results of any other analyses performed that could be used to inform the future definitive trial |
| Harms | 19 | Harms or unintended effects in each group (for specific guidance see CONSORT for harms). If relevant, other important unintended consequences |
| Discussion  Limitations | 20 | Pilot trial limitations, addressing sources of potential bias and remaining uncertainty about feasibility |
| Generalisability | 21 | Generalisability (applicability) of pilot trial methods and findings to future studies |
| Interpretation | 22 | Interpretation consistent with pilot trial objectives and findings, balancing potential benefits and harms, and considering other relevant evidence |
|  | 22a | Implications for progression from pilot to future definitive trial |
| Other information: Registration, protocol ID, funding and ethical approval | | |

*CONSORT

Finally, while the proposed scoping review is not designed to assess the efficacy of interventions, it may be especially important to map the existing practice when reporting outcomes and measures used, as these may affect the feasibility and acceptability of the study methods. Thus, any measure will be considered relevant to this scoping review (Table 7), as we aim to create a comprehensive overview of the psychoeducational group interventions.

**Table 7**

**Table for data extraction for PROM and PREM**

Note: * Authors reported the scale was validated among ADHD

| **Study ID** |  |
| --- | --- |
| Variables reported  at baseline |  |
| Reported at post-  Or follow-up |  |
| ADHD |  |
| Skills |  |
| Measures of knowledge |  |
| Self-rated scales not validated in ADHD |  |
| QoL |  |
| Patient satisfaction |  |
| Self-efficacy and/or self-esteem |  |
| Other PROM or PREMs |  |

Following the data charting, we aim to conduct a narrative synthesis of the included studies by summarizing the findings related to feasibility, acceptability, and outcome measures to identify the patterns, gaps, and trends in the existing literature.

**RESULTS**

We aim to present the results in their entirety in one or more tables that map out the relevant information, as well as in the form of narrative summaries that further describe how the results align with the scoping review objectives, thereby facilitating a comprehensive overview of the findings. It is anticipated that the evidence will initially be categorized into: evidence focused on the feasibility of the interventions, acceptability, outcomes used in such interventions in the context of adult ADHD, and whether its validity and reliability were tested.

**DISCUSSION AND CONCLUSION**

This is a study protocol for a scoping review that aims to comprehensively examine the feasibility, acceptability, and outcomes used in psychoeducational group interventions in the context of adult ADHD. This scoping review will be the first to synthesize the existing evidence in such interventions, using an innovative approach by involving a user representative in designing this protocol. User involvement will be important throughout the project, including in the determination of the objectives and improving the lay interpretability of the review.

The potential limitations of this review include language bias due to including only English-language publications. The identification of feasibility and acceptability issues could pave the way for the development of psychoeducational group interventions and may provide a roadmap for researchers to explore the effectiveness of these interventions. Summarizing the emerging research in this area will provide important insights into the approaches and outcomes being used, the gaps and limitations of this work, and avenues for future research. The results will be used to inform the further adaptation of a psychoeducational group intervention targeting adults newly diagnosed with ADHD. Such findings will be useful for implementation considerations, including mental-healthcare providers delivering psychoeducational group interventions.

To conclude, this protocol establishes the background and methodological foundation for a scoping review that will serve as a critical initial step in advancing the understanding of psychoeducational group interventions. The findings of the scoping review will assist researchers, user representatives, and clinicians in the further establishment of psychoeducational group interventions.

**ETHICS AND DISSEMINATION**

No ethical approval or informed consent will be required, as no original data will be collected. Results will be published in an open-access, peer-reviewed journal, presented at academic conferences, and disseminated among lay and professional healthcare audiences.

**FUNDING STATEMENT AND COMPETING INTEREST**

TS was supported by Ph.D. grants from the Central Norway Regional Health Authority. The authors who will contribute to the scoping review have no competing interests to report.

**REFERENCES**

Beentjes, T. A. A., van Gaal, B. G. I., van Achterberg, T., & Goossens, P. J. J. (2020). Self-Management Support Needs From the Perspectives of Persons With Severe Mental Illness: A Systematic Review and Thematic Synthesis of Qualitative Research. *J Am Psychiatr Nurses Assoc*, *26*(5), 464-482. <https://doi.org/10.1177/1078390319877953>

Biederman, J., Faraone, S. V., Spencer, T. J., Mick, E., Monuteaux, M. C., & Aleardi, M. (2006). Functional impairments in adults with self-reports of diagnosed ADHD: A controlled study of 1001 adults in the community. *J Clin Psychiatry*, *67*(4), 524-540. <https://doi.org/10.4088/jcp.v67n0403>

Biederman, J., Fried, R., DiSalvo, M., Woodworth, K. Y., Biederman, I., Driscoll, H., Noyes, E., Faraone, S. V., & Perlis, R. H. (2020). Further evidence of low adherence to stimulant treatment in adult ADHD: an electronic medical record study examining timely renewal of a stimulant prescription. *Psychopharmacology (Berl)*, *237*(9), 2835-2843. <https://doi.org/10.1007/s00213-020-05576-y>

Ekhtiari, H., Rezapour, T., Aupperle, R. L., & Paulus, M. P. (2017). *Neuroscience-informed psychoeducation for addiction medicine: A neurocognitive perspective* (Vol. 235). <https://doi.org/10.1016/bs.pbr.2017.08.013>

Fullen, T., Jones, S. L., Emerson, L.-M., & Adamou, M. (2020). Psychological Treatments in Adult ADHD: A Systematic Review. *Journal of Psychopathology and Behavioral Assessment*, 1-19.

Gjervan, B., Torgersen, T., Nordahl, H. M., & Rasmussen, K. (2012). Functional impairment and occupational outcome in adults with ADHD. *J Atten Disord*, *16*(7), 544-552. <https://doi.org/10.1177/1087054711413074>

Greene, J. A., Bina, R., & Gum, A. M. (2016). Interventions to Increase Retention in Mental Health Services: A Systematic Review. *Psychiatr Serv*, *67*(5), 485-495. <https://doi.org/10.1176/appi.ps.201400591>

Hafstad, E., & Leiknes, K. A. (2016). NIPH Systematic Reviews: Executive Summaries. In *Psychoeducation for Adults with Attention Deficit Hyperactivity Disorder (ADHD): Rapid Review*. Knowledge Centre for the Health Services at The Norwegian Institute of Public Health (NIPH)

Copyright © 2016 by The Norwegian Institute of Public Health (NIPH).

Hoffmann, T. C., Glasziou, P. P., Boutron, I., Milne, R., Perera, R., Moher, D., Altman, D. G., Barbour, V., Macdonald, H., Johnston, M., Lamb, S. E., Dixon-Woods, M., McCulloch, P., Wyatt, J. C., Chan, A. W., & Michie, S. (2016). [Better Reporting of Interventions: Template for Intervention Description and Replication (TIDieR) Checklist and Guide]. *Gesundheitswesen*, *78*(3), e174. <https://doi.org/10.1055/s-0037-1600948> (Die TIDieR Checkliste und Anleitung – ein Instrument für eine verbesserte Interventionsbeschreibung und Replikation.)

Katzman, M. A., Bilkey, T. S., Chokka, P. R., Fallu, A., & Klassen, L. J. (2017). Adult ADHD and comorbid disorders: clinical implications of a dimensional approach. *BMC Psychiatry*, *17*(1), 302. <https://doi.org/10.1186/s12888-017-1463-3>

Keen, A., Lu, Y., Oruche, U. M., Mazurenko, O., & Draucker, C. B. (2021). Activation in persons with mental health disorders: An integrative review. *J Psychiatr Ment Health Nurs*, *28*(5), 873-899. <https://doi.org/10.1111/jpm.12789>

Kelly, E. L., Fenwick, K. M., Barr, N., Cohen, H., & Brekke, J. S. (2014). A systematic review of self-management health care models for individuals with serious mental illnesses. *Psychiatr Serv*, *65*(11), 1300-1310. <https://doi.org/10.1176/appi.ps.201300502>

Leitan, N. D., Michalak, E. E., Berk, L., Berk, M., & Murray, G. (2015). Optimizing delivery of recovery-oriented online self-management strategies for bipolar disorder: a review. *Bipolar Disord*, *17*(2), 115-127. <https://doi.org/10.1111/bdi.12258>

Newark, P. E., Elsässer, M., & Stieglitz, R.-D. (2016). Self-Esteem, Self-Efficacy, and Resources in Adults With ADHD. *Journal of Attention Disorders*, *20*(3), 279-290. <https://doi.org/10.1177/1087054712459561>

Newark, P. E., & Stieglitz, R.-D. (2010). Therapy-relevant factors in adult ADHD from a cognitive behavioural perspective. *ADHD attention deficit and hyperactivity disorders*, *2*(2), 59-72. <https://doi.org/10.1007/s12402-010-0023-1>

Nimmo-Smith, V., Merwood, A., Hank, D., Brandling, J., Greenwood, R., Skinner, L., Law, S., Patel, V., & Rai, D. (2020). Non-pharmacological interventions for adult ADHD: a systematic review. *Psychol Med*, *50*(4), 529-541. <https://doi.org/10.1017/s0033291720000069>

Oliveira, C. T. d., & Dias, A. C. G. (2018). Psychoeducation for attention deficit/hyperactivity disorder: what, how and who shall we inform? *Trends in Psychology*, *26*, 243-261.

Peters, M. D. J., Marnie, C., Tricco, A. C., Pollock, D., Munn, Z., Alexander, L., McInerney, P., Godfrey, C. M., & Khalil, H. (2020). Updated methodological guidance for the conduct of scoping reviews. *JBI Evid Synth*, *18*(10), 2119-2126. <https://doi.org/10.11124/jbies-20-00167>

Pollock, D., Alexander, L., Munn, Z., Peters, M. D. J., Khalil, H., Godfrey, C., McInerney, P., Synnot, A. J., & Tricco, A. C. (2022). Moving from consultation to co-creation with knowledge users in scoping reviews: guidance from the JBI Scoping Review Methodology Group. *JBI Evidence Synthesis*, *20*, 969 - 979.

Sandra, M. E., Claire, L. C., Michael, J. C., Christine, M. B., Sally, H., Lehana, T., & Gillian, A. L. (2016). CONSORT 2010 statement: extension to randomised pilot and feasibility trials. *Bmj*, *355*, i5239. <https://doi.org/10.1136/bmj.i5239>

Scholz, L., Werle, J., Philipsen, A., Schulze, M., Collonges, J., & Gensichen, J. (2020). Effects and feasibility of psychological interventions to reduce inattention symptoms in adults with ADHD: a systematic review. *Journal of Mental Health*, 1-14. <https://doi.org/https://dx.doi.org/10.1080/09638237.2020.1818189>

Seery, C., Wrigley, M., O'Riordan, F., Kilbride, K., & Bramham, J. (2022). What adults with ADHD want to know: A Delphi consensus study on the psychoeducational needs of experts by experience. *Health Expect*, *25*(5), 2593-2602. <https://doi.org/10.1111/hex.13592>

Siantz, E., & Aranda, M. P. (2014). Chronic disease self-management interventions for adults with serious mental illness: a systematic review of the literature. *Gen Hosp Psychiatry*, *36*(3), 233-244. <https://doi.org/10.1016/j.genhosppsych.2014.01.014>

Solberg, B. S., Haavik, J., & Halmoy, A. (2019). Health Care Services for Adults With ADHD: Patient Satisfaction and the Role of Psycho-Education. *Journal of Attention Disorders*, *23*(1), 99-108. <https://doi.org/https://dx.doi.org/10.1177/1087054715587941>

Song, P., Zha, M., Yang, Q., Zhang, Y., Li, X., & Rudan, I. (2021). The prevalence of adult attention-deficit hyperactivity disorder: A global systematic review and meta-analysis. *J Glob Health*, *11*, 04009. <https://doi.org/10.7189/jogh.11.04009>

Torgersen, T., Gjervan, B., & Rasmussen, K. (2006). ADHD in adults: a study of clinical characteristics, impairment and comorbidity. *Nord J Psychiatry*, *60*(1), 38-43. <https://doi.org/10.1080/08039480500520665>

Tricco, A. C., Lillie, E., Zarin, W., O'Brien, K. K., Colquhoun, H., Levac, D., Moher, D., Peters, M. D. J., Horsley, T., Weeks, L., Hempel, S., Akl, E. A., Chang, C., McGowan, J., Stewart, L., Hartling, L., Aldcroft, A., Wilson, M. G., Garritty, C., . . . Straus, S. E. (2018). PRISMA Extension for Scoping Reviews (PRISMA-ScR): Checklist and Explanation. *Annals of Internal Medicine*, *169*(7), 467-473. <https://doi.org/10.7326/m18-0850>

Zalsman, G., & Shilton, T. (2016). Adult ADHD: A new disease? *Int J Psychiatry Clin Pract*, *20*(2), 70-76. <https://doi.org/10.3109/13651501.2016.1149197>

**Figure 1.**

Reports assessed for eligibility (n = )

Reports sought for retrieval (n = )

Reports not retrieved (n = )

Records excluded** (n = )

Records screened (n = )

**Screening**

**Identification**

**Identification of studies via databases and registers**

Records identified from *: Databases (n = ) Registers (n = )

Records removed *before screening*:

Duplicate records removed (n = )

Records marked as ineligible by automation tools (n = ) Records removed for other reasons (n = )

Studies included in review (n = )

Reports of included studies

(n = )

Reports excluded: Reason 1 (n = ) Reason 2 (n = ) Reason 3 (n = ) etc.

**Included**
